# Exploring the Synergistic Effects of Ultrafine Polyaniline Nanofibers and Oxygen-Modified Multi-Walled Carbon Nanotubes on Enhancing Pseudocapacitive Electrochemical Performance for Advanced Supercapacitors

**DOI:** 10.3390/ma19071356

**Published:** 2026-03-29

**Authors:** Fahima Djefaflia, Ouanassa Guellati, Assia Nait Merzoug, Aicha Harat, Jamal El Haskouri, Izabela Janowska, Mihaela Baibarac

**Affiliations:** 1 Laboratoire d’Etude et de Recherche des Etats Condensés (LEREC), Physic Department, University of Annaba, BP. 12, Annaba 23000, Algeria; abenlala@yahoo.fr (A.N.M.); harat_aicha@yahoo.fr (A.H.); 2 Faculty of Science and Technology, University of Souk Ahras, BP. 1553, Souk-Ahras 41000, Algeria; 3 Instituto de Ciencias de los Materiales, Universitat de València, C/Catedrático José Beltrán, 2, 46980 Paterna, Valencia, Spain; jamal.haskouri@uv.es; 4 Institut de Chimie et Procédés Pour l’Énergie, l’Environnement et la Santé (ICPEES), CNRS UMR 7515, Université de Strasbourg, 25 Rue Becquerel, 67087 Strasbourg, France; janowskai@unistra.fr; 5 National Institute of Materials Physics, Atomistilor Street, No 405 A, 077125 Magurele, Romania

**Keywords:** polyaniline, functionalized carbon nanotube (O-CNT), nanofiber, nanocomposites, electrochemical behavior, energy storage

## Abstract

This work reports a systematic study concerning the synthesis of pure polyaniline ultrafine nanofibers (PANI-NFs) and their nanocomposites with oxygen-functionalized carbon nanotubes (PANI-NFs/O-MWCNTs) using diluted chemical polymerization and hydrothermal processes. We investigated the synergistic effects of various synthesis parameters, such as the concentration of the ammonium persulfate oxidant agent and growth temperature, on the physical, chemical, and electrochemical properties of the resulting products through structural, morphological, spectroscopic, and electrochemical characterization. Our study revealed the successful synthesis of thermally resistant polyaniline ultrafine nanofibers (PANI-NFs) in the form of emeraldine salt (ES), exhibiting a mean diameter in the range of 8–17 nm. The PANI-NFs and PANI-NFs/O-MWCNT nanocomposites demonstrated excellent electrochemical properties, with specific capacitances of up to 0.94–1.23 F cm^−2^ and 1410–2074 F/g, respectively, and with good rate capability. These characteristics are confirmed by the relaxation time constant τ_0_ (41 and 8 ms, respectively) and lower internal R_0_/interfacial charge transfer R_Փ_ resistances of around 0.2 Ω, as well as diffusion coefficients of around 10^−7^ and 3.7 × 10^−7^ cm^2^/s. This breakthrough in nanofiber synthesis paves the way for practical applications in diverse domains, from high-performance energy storage to biosensing and beyond, where the unique electroactive properties of the nanocomposites can be leveraged to achieve exceptional results.

## 1. Introduction

Advanced innovative research on electroactive nanomaterials such as carbon, transition metal oxides/hydroxides, and conducting polymers has made significant strides in recent years. These materials have been shown to have widespread potential applications, including in chemical sensors, catalytic supports, corrosion protection, environmental protection, solar cells, and energy storage [1,2,3]. Notably, supercapacitors have gained significant attention due to their high power density, fast charge–discharge rates, and exceptional cyclic stability, positioning them as promising electrochemical energy storage (EES) devices [4,5,6]. Traditionally, the energy storage behavior of supercapacitors is generally classified into two main categories: (1) electric double-layer capacitance (EDLC), which is the result of the pure electrostatic attraction between ions and the charged porous surface of carbon-based materials, and (2) pseudocapacitance (redox), which is attributed to Faradaic reactions between the electrolyte ions and the electroactive components of the electrode, such as conducting polymers or transition metal oxides/hydroxides [7,8,9,10,11,12,13]. These distinctions in energy storage mechanisms provide crucial insight into the optimization of supercapacitor performance.

In addition to their unique and remarkable properties, carbon nanostructures such as carbon nanotubes (CNTs, including multi-walled carbon nanotubes (MWCNTs)), graphene, and activated carbon have been recognized as highly promising electroactive nanomaterials for use in the development of electric double-layer capacitance (EDLC) supercapacitors [8,14,15,16,17]. Among these nanostructures, MWCNTs are particularly noteworthy due to their unique characteristics that make them ideal for electrochemical applications: their remarkable specific surface area, outstanding electrical conductivity, impressive mechanical properties, and exceptional chemical stability. However, their low specific capacitance currently limits their use in supercapacitor electrodes [18,19].

In contrast to carbon-based nanomaterials, conducting polymers (CPs) such as polyaniline (PANI), polypyrrole (PP), and polythiophene (PT) have also been extensively studied as potential electroactive materials in electrochemical redox-based supercapacitors. Among these CPs, PANI has emerged as a promising candidate due to its unique properties, such as specific capacitance, low-cost and simple synthesis, high conductivity, and excellent environmental and thermal stability [20,21,22]. Polyaniline (PANI) exists in three oxidation states: fully oxidized (pernigraniline: blue), fully reduced (leucoemeraldine: colorless), and partially oxidized (emeraldine base: violet or emeraldine salt: green). Of these, emeraldine salt is the most crucial form, boasting remarkable conductivity (~100 S·cm^−1^), which facilitates its application in energy storage devices, particularly supercapacitors [23,24,25,26,27]. However, PANI suffers from limited cycling stability due to its tendency to swell and shrink during charging and discharging cycles, coupled with reduced electroactivity in neutral pH conditions.

To address these drawbacks, researchers have recently explored integrating PANI with carbon or metal-based nanomaterials, leveraging the beneficial properties of each component to enhance the electrochemical characteristics of PANI and its application in energy storage devices [28,29,30,31,32,33,34]. B.P. Prasanna et al. synthesized PANI/MWCNTs as a binary electrode nanocomposite by using oxidative interfacial polymerization and obtained a specific capacitance of 155 F·g^−1^ at 2 mV·s^−1^ in 1M H_2_SO_4_ electrolyte with a retention of 95% over 1000 cycles [35]. Das et al. reported the synthesis of NiO/PANI-MWCNT ternary nanocomposites via an in situ polymerization method for a high-performance supercapacitor electrode with a specific capacitance of 356.54 F·g^−1^ at 5 mV·s^−1^ [36]. In addition, the synthesis of CuO@NiO/polyaniline/MWCNT quaternary nanocomposites using chemical methods has been reported by Chakraborty et al. [37]. Their electrochemical measurements exhibited an excellent specific capacitance of 1372 F·g^−1^, with good cycling stability (retaining 83% after 1500 cycles). Cao and co-workers reported the synthesis of layered double hydroxide (LDH)/PANI for supercapacitor applications by using in situ polymerization, and they showed a good specific capacitance of 592 F·g^−1^ and high retention of 87% over 500 cycles [28].

Our investigations describe a new method for synthesizing ultrafine nanoscale polyaniline (PANI) and its nanocomposites (PANI/O-MWCNTs), which had not previously been achieved, using cost-effective diluted chemical and hydrothermal methods without templates. We conducted a thorough study of the effects of key synthesis parameters, including oxidant type and concentration, growth method, and hydrothermal growth temperature, on the structural, textural, morphological, spectroscopic, and electrochemical properties of these PANI-based nanomaterials. Our results demonstrated a remarkable improvement in the electrochemical performance of the PANI/O-MWCNT nanocomposite compared with the pure PANI-based supercapacitor electrode, based on electrochemical measurements performed in a KOH aqueous electrolyte using a three-electrode configuration. This outcome is particularly significant because this specific configuration has not been widely explored in the literature.

## 2. Materials and Methods

Aniline monomer (C_6_H_5_NH_2_, purity ≥ 99%), hydrochloric acid (HCl, 35% purity), and ammonium persulfate (APS, (NH_4_)_2_S_2_O_8_, 98% purity) were purchased from Sigma-Aldrich (Saint-Quentin-Fallavier, France). All the chemical precursors were analytical-grade and used without further purification. Nickel foam (NiF) was purchased from Alantum (Munich, Germany) with an areal density of 420 g·m^−2^, a diameter of 1.6 mm, and a thickness of 0.2 mm. The multi-walled carbon nanotubes (MWCNTs) used in this study were synthesized using a catalytic-CVD method at a growth temperature of 750 °C under atmospheric pressure, with ethylene as the carbon source and nitrogen as the carrier gas, over an Fe/Al_2_O_3_ support catalyst [38]. The MWCNTs were oxidized via an acidic treatment with a mixture of hydrochloric acid and nitric acid (1:3 ratio) at 70 °C for 2 h. Following this, they were thoroughly washed with deionized water and filtered until the pH of the solution was neutral. The functionalized MWCNTs (O-MWCNTs) were then dried at 80 °C for 20 h. These O-MWCNTs have a specific surface area of approximately 140 ± 0.4425 m^2^·g^−1^.

### 2.1. PANI Ultrafine Nanofiber (PANI-NF) and PANI-NF/O-MWCNT Synthesis

#### 2.1.1. Chemical Process (DCP)

First, 40 mM aniline monomers and 20 mM HCl as a doping agent were mixed and magnetically stirred for 30 min. Then, 30 mL of distilled water (or DI with dispersed O-MWCNTs “1 g/L” in the case of the nanocomposite for in situ polymerization) was added to the above mixture under vigorous magnetic stirring at room temperature for 15 min. After that, the obtained light pink solution was maintained inside an ice bath (˂5 °C) under constant vigorous magnetic stirring; the oxidant APS (1 mM) (or ultrasonicated APS/O-MWCNTs) was added after around 5 min to start the polymerization/solution color change, and the reactions were followed for 5 h. The polymerization was complete when the characteristic green color of the PANI emeraldine salt form was produced (see the schematic below). Afterwards, the resulting dark green precipitates were filtered and washed several times with distilled water and ethanol to remove any residual acid. Finally, the products were dried in an oven at 70 °C overnight. The samples in the figures are labeled as follows: DCP (dilution chemical polymerization process), HT (hydrothermal process), CP/CNTs (CP for PANI/O-MWCNTs), and HT/CNTs (HT for PANI/O-MWCNTs).

#### 2.1.2. Free-Template Hydrothermal Process (HT)

**Figure 1:**
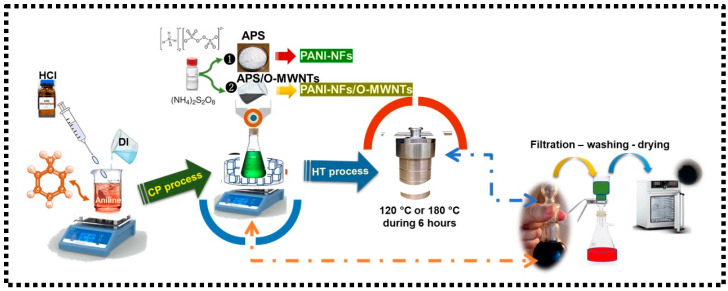
Chemical and hydrothermal process for simultaneous doping–polymerization of PANI-NFs and PANI-NFs/CNTs.

In this simple and low-cost synthesis process, the obtained solutions from the preceding process (DCP) with a dark green color were transferred into a 40 mL Teflon-lined hydrothermal autoclave system (Constantine, Algeria), which was sealed and maintained at two different growth temperatures (120 and 180 °C) for 6 h (Figure 1). After that, the system was left to cool down naturally to room temperature. The resulting green products were filtered and sequentially washed several times with distilled water and ethanol before drying in an oven at 70 °C for 24 h.

### 2.2. Sample Characterization

The crystal structures of the synthesized pure PANI and PANI/O-MWCNT nanocomposites were characterized using an XRD D8 ADVANCE AXS diffractometer (BRUKER, Berlin, Germany) equipped with CuKα radiation and a graphite monochromator rear blade, operated at 40 kV and 40 mA with a step size of 0.2°·s^−1^ in the range of 10° to 90°.

The chemical composition and spectroscopic characteristics of the as-synthesized materials were analyzed using Fourier-Transform Infrared (FTIR) and Raman spectroscopy. FTIR spectra were collected on a Bruker Vertex 77v spectrometer in the range of 400 to 4000 cm^−1^ with a resolution of 4 cm^−1^. Raman scattering measurements were performed on a Horiba Jobin Yvon Lab-RAM Aramis confocal Raman spectrometer at room temperature, equipped with a cooled CCD camera and an automated XYZ table. The spectrometer utilized an excitation wavelength of 532 nm, with a power of 0.33 mW reaching the samples, and was equipped with a D2 filter. These experimental conditions ensured the acquisition of high-quality Raman spectra, which provided valuable and crucial information about the vibrational characteristics of the samples.

The synthesized products were morphologically and texturally characterized using Field Emission Scanning Electron Microscopy (FE-SEM) on a JEOL 6700-FEG microscope (Akishima City, Tokyo) at an acceleration voltage of 3 kV. However, High-Resolution Transmission Electron Microscopy (HRTEM) performed with the JEOL-JEM-1010 instrument operated at 100 kV (equipped with the AMT XR80 digital camera MegaView III) allowed the visualization of the samples at high magnifications and resolutions.

The thermal stability of the samples was investigated by thermogravimetric analysis (TGA) using a TGA 550-TA instrument (New Castle, DE, USA). The weight loss and derivative weight of the samples were measured by heating them from room temperature to 1000 °C at a rate of 10 °C/min in an air atmosphere. These measurements provided information on the decomposition of the materials at elevated temperatures, as well as the temperatures at which weight loss occurs, which are critical parameters for evaluating the stability and durability of the samples.

The surface chemistry of the synthesized PANI and PANI/O-MWCNT nanocomposites was further examined by X-ray photoelectron spectroscopy (XPS), which allowed for the identification of the chemical bonds present in the materials. A Thermo Scientific spectrometer with Al Kα radiation (1486.6 eV) and a hemispherical analyzer was used to acquire XPS spectra. A broad survey scan from 0 to 1400 eV was conducted to determine the dominant elements in the samples, followed by a calibration based on the graphitic carbon C1s level fixed at 284.6 eV. The main peaks were further deconvoluted using a Gaussian/Lorentzian function (30% weighting) in the Avantage software 5.27 provided by Thermo-electron Company (United States), based on results reported in the literature. This deconvolution enabled us to obtain more precise information about the surface chemistry and chemical bonding in the PANI-based products, which is essential for understanding their properties and performance in supercapacitor applications.

### 2.3. Working Electrode Preparation and Electrochemical (EC) Measurements

The electrodes of the supercapacitors with PANI and PANI/O-MWCNT nanocomposites as the active material were prepared by mixing PANI (with or without O-MWCNTs) with carbon black (CB), along with polyvinylidene difluoride (PVDF) as a binder, in a weight ratio of 80:10:10 (wt.%), with a few drops of 1-methyl-2-pyrrolydone (NMP) as the solvent. The mixture was homogenized in an agate mortar to form a paste, which was then coated onto a 1 cm × 1 cm pre-treated nickel foam (NiF) current collector. After that, the electrodes with an electroactive mass loading of 1.0–1.5 mg·cm^−2^ were dried at 80 °C for 24 h to remove residual NMP and ensure strong adhesion and good conductivity. The exact loading used for each electrode was as follows: DCP-2%APS, 1 mg; DCP-10%APS, 1,3 mg; HT-120 °C, 1 mg; HT-180 °C, 1,2 mg; DCP/CNTs, 1,3 mg; HT/CNTs-120 °C, 1 mg; and HT/CNTs-180 °C, 1 mg.

The electrochemical performance was evaluated using a Gamry R600 workstation (REF600 TM Potentiostat/Galvanostat/ZRA) at room temperature in a three-electrode configuration. The prepared PANI or PANI/O-MWCNT electrodes served as the working electrodes; platinum was used as the counter electrode, and Hg/HgO (3M KCl) acted as the reference electrode. All tests were performed in an aqueous 6M KOH electrolyte.

Cyclic voltammetry (CV) was carried out in the potential window of 0.0–0.6 V at scan rates ranging from 5 to 100 mV·s^−1^; galvanostatic charge–discharge (GCD) tests were performed at current densities between 1 and 100 A·g^−1^; and electrochemical impedance spectroscopy (EIS) measurements were obtained at open-circuit potential over the frequency range of 0.01–100 kHz to analyze the charge-transfer resistance and ion-diffusion characteristics.

Consequently, the electrochemical performance of these synthesized samples was quantitatively evaluated in terms of their areal capacitance (Cs in F·cm^−2^) from the CV curves and the specific capacitance (Qs in F·g^−1^) from the GCD curves using the following equations:

(CV curve): C_s_ = (∫IdV)/mνΔV (1)

(GCD curve): Q_s_ = iΔt/mΔV (2)

where m is the loaded active material weight (g), namely, the mass of only the active material excluding the binder and conductive agent; ν corresponds to the scan rate (mV·s^−1^); ΔV (Vc − Va) is the potential window; ∫IdV is the integrated area under the CV curve for the cathodic current region (mA·V) in Equation (1); I = i/m (A/g) is the current density in Equation (2); Δt (s) is the discharge time; and ΔE is the voltage difference in discharge, eliminating the section of IR drop.

## 3. Results and Discussion

### 3.1. Optical and Structural Properties of PANI-NF and the PANI/O-MWCNT Nanocomposites

To investigate the effective parameters that influence the structural/textural and morphological properties of the produced PANI-based conductive nanocomposites, as well as their electrochemical behavior and characteristics, various conditions of the oxidative DCP in a diluted strong acidic solution were applied and optimized. The parameters studied include the APS/ANI precursor ratio, the growth method, the hydrothermal growth temperature, and the O-MWCNT reinforcement.

**Figure 2:**
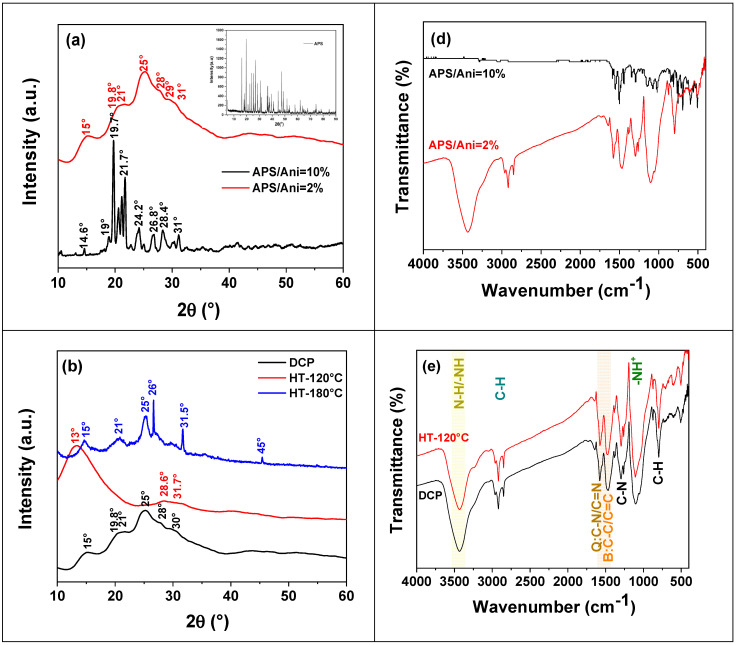

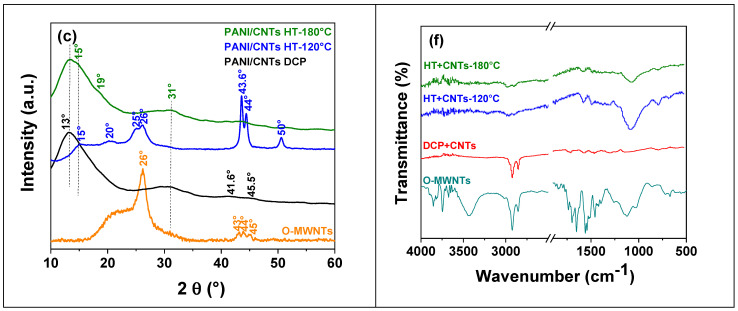
XRD and FTIR spectra of PANI NFs when the APS/Ani ratio is equal to 2 and 10 (**a**,**d**), and synthesis method (**b**,**e**), as well as the effect of incorporating O–MWCNTs on the structural and binding properties of the obtained PANIs (**c**,**f**).

First, the structural properties regarding product selectivity and purity were studied using XRD (Figure 2). Figure 2a illustrates the crystallographic identification of the obtained pure PANI prepared via the DCP polymerization method with a precursor/monomer (APS/ANI) ratio of 2%. These patterns exhibit four main broad peaks in the range of 10–35°, which confirms the formation of conductive polyaniline type emeraldine salt (ES) in semi-amorphous form with a considerable level of structural ordering, as reported previously by several studies [39,40,41,42]. The predominant peak around 19.8°, associated with the (020) crystal plane of PANI, could be attributed to the parallel periodicity of the polymer chains [39,43].

However, with a higher amount of the polymerization oxidant APS (APS/ANI = 10%), the XRD profile clearly shows four broad peaks at approximately 14.6°, 19.7°, 21.7°, and 31° (d spacings = 0.61, 0.45, 0.41, and 0.29 nm), corresponding to the crystalline planes (011), (020), (100), and (211) of PANI in the semi-crystalline emeraldine salt form, as also reported in several studies [17,35,41,44,45,46]. Moreover, the XRD results show a broad peak phase with lower crystallinity (~33%) for pure PANI prepared with lower APS content, whereas with an increasing amount of APS, the as-synthesized pure PANI has a higher density of structural arrangement in sharp peaks, reaching a crystallinity degree of around 51%. Furthermore, a pure APS XRD diagram shows sharp diffraction peaks, which illustrate an obvious crystal structure, as reported by Y. Gong [47] and B. Qiu [48]. Nevertheless, with the HT process at two different growth temperatures, the XRD pattern of pure PANI is considerably different from that obtained in the first-step synthesis via the DCP process (Figure 2b). After synthesis at 120 °C for 6 h, two main broad peaks were observed around 13° and 28.6°, which were assigned to the (010) and (211) crystal planes, respectively [41,49]. Furthermore, with growth at 180 °C for 6 h, the PANI pattern shows the appearance of three sharp peaks at 14.84°, 20.77°, and 25.49°, assigned to the PANI emeraldine salt form, confirming the presence of benzenoid and quinonoid rings in PANI, as reported above, but with improved crystallinity [44]. The last two peaks are assigned to the periodicities parallel and perpendicular, respectively, to the PANI chains [39,41,50]. The hydrothermal process, with its conditions of pressure and growth temperature, caused the PANI chains to rearrange and form a more ordered structure at 180 vs. 120 °C. Moreover, the produced PANI structure varied from amorphous to partially crystalline depending on the polymer doping/protonation state, which is expected to exhibit high electrical conductivity and will be further identified via spectroscopic analysis. In the case of PANI/MWCNT nanocomposites (Figure 2c), the same peaks of pure PANI prepared by the HT process were also indexed, proving good interactions between PANI-ES and O-MWCNTs without changes in either. It is therefore clear that no additional order was introduced into the nanocomposites, and their crystallinity has not improved in comparison with pure PANI. Furthermore, the presence of MWCNTs was confirmed through four crystalline peaks at 26.06° (overlapping with the peak from PANI), 43.58°, 44.47°, and 50.65°, corresponding to the (002), (100), (101), and (102) crystalline planes, respectively [34,35,49,50]. Consequently, all the above results demonstrate the successful synthesis of the PANI/MWCNT nanocomposite via the hydrothermal method, with greater crystallinity and a highly ordered chain structure of the conducting polymer, which induces strong electrical conductivity, as reported by previous studies in different fields.

To better elucidate the changes taking place in PANI/MWCNT nanocomposites and to evaluate their molecular configuration, FT-IR spectroscopy was performed (Figure 2d–f). In general, pure PANI exhibits absorption peaks around 1552 cm^−1^ and 1490 cm^−1^, corresponding to the vibrational modes of the stretching of C=C bonds in the quinoid (Q) ring and benzene (B) ring, respectively, in undoped PANI [49,51]. The degree of PANI oxidation (x) could be estimated via the following equation [51,52,53]:

(3) $$x=\frac{\left(I_{Q}/I_{B}\right)}{1+I_{Q}/I_{B}}$$

in which *I_Q_/I_B_* represents the intensity ratio of quinoid (Q) and benzenoid (B) rings, found between 1 and 1.16 due to enhanced conjugation interactions and the doping of the PANI chains with HCl [49]. Therefore, we found x to be between 0.5 and 0.54, proving a low degree of oxidation; the longer the conjugation length, the higher the electrical conductivity. Figure 2d shows the obtained bands for pure PANI prepared with different APS/ANI precursor ratios. The PANI shows a peak at 1580 cm^−1^, assigned to the vibrational mode C–C stretching in the polaronic structure (B–NH^+•^-), as reported previously [54,55,56], while two characteristic peaks emerging at 1471 and 1448 cm^−1^ are attributed to the C–C and C=C stretching vibrational modes of the benzenoid unit (-NH–B–NH-) of PANI [55,56,57,58]. The absorption bands at 1300 and 1295 cm^−1^ are due to the C–N stretching vibration in the secondary aromatic ring and the C–N^+^ stretching mode in the polaronic structure of protonated PANI [48,50,51,57]. The intense broad peak at 1105–1148 cm^−1^, which is more intense in the sample with low APS (2%), is related to the –NH^+^= structure; its presence confirms a high degree of electron delocalization in the structure [49]. This arises due to the vibrational mode of (B–NH^+^=Q) or (B-NH^+^-B) states or N-Q-N-Q stretching, and it is characteristic of the doped ES form [54,57,58]. The appearance of peaks at 1043–1075 cm^−1^ is attributed to the C–H stretching mode of N,N’-diphenyl-1,4-phenylenediamine entities in PANI [59]. In addition, it is generally accepted that the absorption peaks appearing below 1000 cm^−1^ correspond mainly to the planar C–H deformation vibrations of p-disubstituted benzene ring intermediates formed during the polymerization of aniline (para-coupling of aniline units) [49,54,56,57,60]. The propagation reaction of the polymerization of the monomer in a linear head-to-tail mode was suggested [57]. The IR bands at 800–852 cm^−1^ and 510–597 cm^−1^ correspond to C–H out-of-plane bending vibrations and C–C bending vibrations, respectively, in aromatic rings [58]. In the case of pure PANI prepared with more APS (10%), we noted the disappearance of a band at 3425 cm^−1^, which can be attributed to the N–H stretching mode of the leucoemeraldine component arising from hydrogen bonding interactions [54,56,57,61,62]. This again emphasizes that PANI formed with more APS has more quinonoid rings and fewer benzenoid rings in the polymer chain [56]. However, peaks at 2916 and 2924–2852 cm^−1^ are attributed to the asymmetric and symmetric C–H stretching vibrations in benzenic rings in PANI [36,49,55], which disappear with higher APS oxidant precursor content. Otherwise, the FTIR spectrum of pure PANI, in this case with high APS, in the region 1600–500 cm^−1^, is almost the same but with a slight shift to higher or lower wavenumbers [47]. Notably, this proves the slight changes in the electronic state of the PANI products, as shown by the XRD crystallinity degree. Therefore, the HT process did not affect the PANI product binding in the FTIR results (Figure 2e), which agrees with the literature.

However, for the PANI/MWCNT nanocomposites prepared via the DCP process, it is clear from Figure 2f that the FTIR spectrum is similar to that of pure PANI but with slight shifts to lower or higher wavenumbers in the main bands’ maxima around 1149, 1302, 1468, and 1573 cm^−1^, corresponding to the vibrational modes of -NH^+^, -C–N bending in the aromatic amine, -C=C stretching in the B ring, and -C=C stretching in the Q ring of PANI-ES [44,50,59,60]. Furthermore, this shift confirms that PANI/O-MWCNT nanocomposites from in situ polymerization have a high degree of conjugation due to the π–π interactions between PANI and O-MWCNTs [35,61]. It can also be observed that the peaks at 3425, 1290, and 1104 cm^−1^ disappeared, and the peaks around 3242 and 1712 cm^−1^, which are due to the O–H stretching vibration in the carboxyl group, appeared, indicating the presence of carboxylic acid groups on the O-MWCNT walls at defect sites [34,41]. Moreover, the intensity of the peaks at 2922 and 2853 cm^−1^ significantly increased, which also confirms the presence of O-MWCNTs in PANI [49]. Consequently, we suggest that there are strong interactions between the carbonyl group of O-MWCNTs and PANI in its ES form, with Q and/or B rings as chemical dopants during the in situ formation of nanocomposites in specific electronic states, which may significantly affect the charge transfer phenomena within PANI as well as between PANI and O-MWCNTs, hence influencing their conductivity and, thus, the nanocomposites’ electrochemical performance [35,49,63]. Furthermore, Potphode recently reported that the structural orientation and charge carrier mobility of PANI in doped and undoped states, according to the material structures and morphologies at the microscopic level, are strongly connected, which subsequently affects their conductivity [50]. Summarizing these changes, the presence of IR bands in the spectral range 1570–1580 cm^−1^, assigned to the vibrational mode of the polaronic structure (B-NH^+•^-), observed in PANI when the APS/Ani ratio is equal to 2 and 10, as well as in the samples labeled HT-120 °C, DCP, DCP+CNT, HT+CNT 120 °C, and HT+CNT 180 °C, indicates the doped state of PANI of the emeraldine salt type.

**Figure 3:**
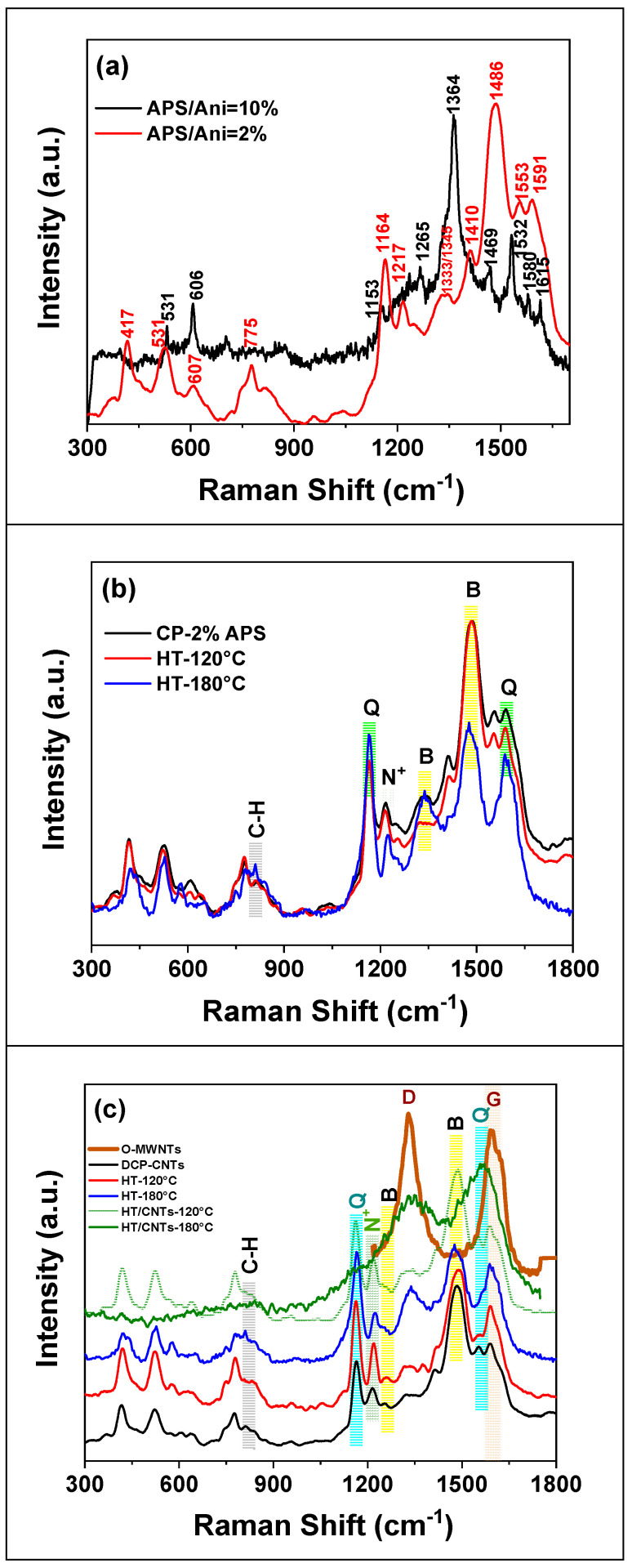
Raman spectra of PANI products depending on APS oxidant (**a**), synthesis method (**b**), and incorporation of O–MWCNTs (**c**).

Further confirmation of these results was sought using Raman spectroscopy. It is also an important tool for checking PANI’s conductivity state from its molecular vibration states and for probing the functional groups and conjugation reactions inherent in the structures of the produced polymers [64]. To evaluate the effect of APS oxidant molarity, Raman spectra were acquired for pure PANI prepared via the DCP process with two different molar ratios of precursors (Figure 3a). In the case of low APS content (APS/Ani = 2%), the spectrum presents typical characteristic bands ascribed to conductive PANI-ES, which appeared at around 1590, 1533–1485, 1410, 1345–1333, 1217, and 1164 cm^−1^. The Raman lines peaking at 1590, 1533–1485, 1410, 1345–1333, 1217, and 1164 cm^−1^ are assigned to the vibrational modes of C–C stretching in the B ring + C=C stretching of the Q ring of PANI-ES [46,65,66], C=N stretching [59], C–C stretching in the Q ring + C–H bending in the Q ring [65,66,67], protonated structures [68,69], C–N stretching, and C–H bending in the Q rings [67,70], respectively. The weak Raman line peaks between 400 and 800 cm^−1^ were owing to the characteristic vibrations of the B and Q rings in the phenazine-like segment [66,68], such as the Q ring deformation at 775 cm^−1^ [70] and B ring deformation at 607 cm^−1^ [60,67], whereas 417 and 531 cm^−1^ correspond to the vibrational modes of C_aromatic_=N of the out-of-plane deformation of the Q ring and deformation of the B ring of PANI [71]. It should be noted that with more oxidant (APS/Ani = 10%), the spectrum exhibits a new strong peak at 1364 cm^−1^ ascribed to the C–NH^+^ vibration of delocalized polaronic structures [72], a sharp peak at 1532 cm^−1^, and a weak one at 1153 cm^−1^. However, we found a decrease in the C=N stretching quinoid ring vibration found around 1469 cm^−1^, with traces of C–C stretching of the benzenoid ring ascribed to 1616 cm^−1^ [66,69]. By contrast, no effect of additional HT treatment or temperature was observed on the PANI Raman spectra, as illustrated in Figure 3b. All the peaks mentioned above also prove the formation of the PANI-ES state via the simultaneous presence of the two benzene and quinone structures.

Figure 3c illustrates several Raman spectra of O-MWCNTs, PANI, and their nanocomposites (PANI/O-MWCNTs). As generally found with CNTs, two major and dominant peaks at 1332 and 1594 cm^−1^ were observed, corresponding to the D and G bands of high-quality O-MWCNTs, where the D band is attributed to disorder and/or defect density in graphitic layers, while the G band is related to sp^2^ hybridized carbon, namely, the “E_2g_ mode” in-plane vibration of the graphitic structure, signifying good crystal purity [45,59,73]. On the other hand, in the PANI/O-MWCNT nanocomposites, we found a combination of the above characteristic peaks of PANI with a clear shift to lower frequencies, accompanied by those of O-MWCNTs with low intensities, confirming the low content of this carbon nanostructure. Therefore, we suggest the existence of strong π–π conjugated interactions between functional groups and the doped PANI molecular chains, which could enhance electron delocalization, cause a Raman shift and consequently facilitate electron transfer at the interface of PANI and O-MWCNT layers. This phenomenon will be confirmed later through EC results [58,59,65,74].

Furthermore, the relative intensity ratio, I_D_/I_G_, is commonly used to estimate defects in graphitic carbonaceous materials, thus indicating the graphitization degree and defect level [39,59]. The obtained I_D_/I_G_ values of the synthesized nanocomposites before and after the HT process are listed in Table 1. The decrease in this intensity ratio confirms the recovery of disorder modified with PANI via π–π conjugation interactions because of the in situ polymerization process followed in this investigation [68]. The above differences and the shifts observed in the various Raman spectrum features indicate the effect of experimental synthesis parameters on the PANI-based nanocomposites with well-incorporated O-MWCNTs, as proved through the interaction between the carboxyl/hydroxyl groups in graphite (O-MWCNTs confirmed from XPS analysis thereafter) and the nitrogen in PANI. This is related to the formation of hydrogen bonding and π–π interactions between graphite sheets and PANI chains, which is consistent with previous reports [59,65,75].

**Figure 4:**
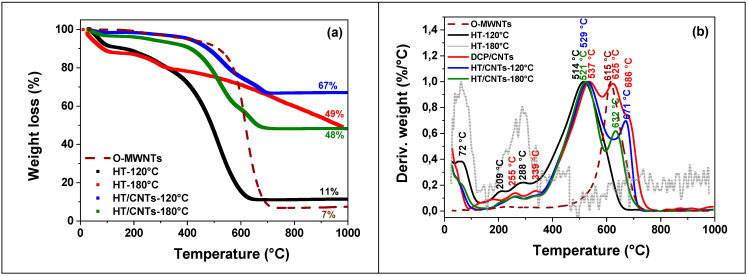
Thermal analysis results of PANI (HT-120 °C, HT-180 °C), O-MWCNTs, and PANI/O–MWCNT nanocomposites with and without the HT process: TG (**a**) and DTA (**b**).

The thermal behavior of PANI, O-MWCNTs, and their nanocomposites was also examined using thermogravimetric analysis (TG-DTA) in an air atmosphere (Figure 4) to evaluate their thermal stability and roughly estimate the oxidation extent and possibility of structural damage. The first weight loss (9%) was observed for pure PANI at 72 °C due to the release of residual moisture and small oligomer molecules, evaporation of solvent, unreacted monomers, and impurities [76]. The second loss was found within a 200–290 °C range (Figure 4a), which can be assigned to the removal of the dopant anion, deprotonation of the PANI salt, and low-molecular-weight oligomers from the polymer backbone [59,76]. The degradation of the PANI backbone units resulted in the formation of substituted aromatic fragments and extended aromatic fragments, as confirmed by bands found around 800 cm^−1^ in the FTIR spectra. Furthermore, the final residue of this pure PANI was around 11 wt.%, with thermal degradation around 514 °C, indicating poor thermal stability due to its semi-amorphous structure, but with higher quality than that reported in the literature [57,59,77,78]. Compared with PANI, O-MWCNTs have better thermal stability and exhibit a sharp weight loss at about 615 °C due to the strong C=C conjugated lattice of the graphitic and highly ordered structure. According to Figure 4a, the MWCNT content is around 14 wt.% in the nanocomposites.

The PANI/O-MWCNT nanocomposites, prepared either via the DCP or DCP and HT processes with two different growth temperatures, showed good stability at higher temperatures, with weight loss (residual) around 33 (67) and 52 (48) wt.% of the total nanocomposite synthesized after the HT step at 120 and 180 °C, respectively. In addition, the thermogram in these cases (Figure 4b) revealed that the products are thermally degraded in three stages, i.e., around 123 °C, 123–243 °C, and 243–700 °C. In the first step, 1–4% weight loss corresponds to moisture and impurities, as well as excess unused aniline monomer; in the second step, 3–4 wt.% loss was due to decomposition via the deprotonation of the PANI salt through the loss of the HCl dopant [40,59,76]. In the last step of this thermal degradation, a sharp decline in the weight of PANI-NFs or their nanocomposites occurred due to the decomposition or substantial disintegration of the polyaniline nanofibers with different degrees of polymerization and functionalized carbon-type O-MWCNTs. The weight decreases to around 30 to 50% by 550–600 °C, suggesting the production of a stable carbon residue [79]. Moreover, the degradation of PANI shifted to higher temperatures in PANI/O-MWCNT nanocomposites compared with pure PANI, thus showing good stability up to 640–680 °C and up to a 70 wt.% yield for the nanocomposite obtained at 120 °C, which represents high-molecular-weight doped chains as mentioned above. Therefore, the combination of PANI and O-MWCNTs with the HT process as an added step could clearly improve the stability of the nanocomposite, where the O-MWCNT nanostructures were uniformly embedded with PANI chains via interfacial interactions [36,76].

**Figure 5:**
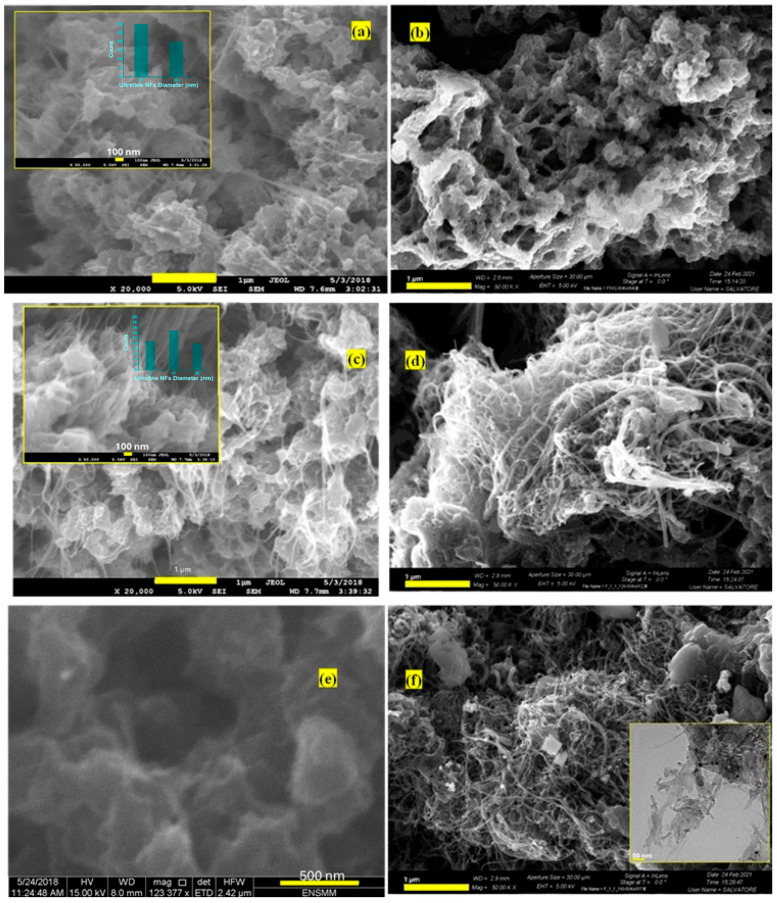
FESEM and HRTEM micrographs of synthesized PANI-NFs with (right) and without (left) O-MWCNTs: DCP (**a**,**b**) and HT process step: 6 h/120 °C (**c**,**d**) and 6 h/180 °C (**e**,**f**).

The morphological aspect, especially in polymers, plays a very important role in their contact area estimation and consequently in their interaction with the application medium. Therefore, in this part, we focus on the morphological–textural properties of the PANI conductor polymer. Figure 5 depicts the FESEM micrographs of pure synthesized PANI and its nanocomposites before (DCP) and after the hydrothermal (HT) process at 120 and 180 °C growth temperatures. The formation of networks of PANI nanofibers (PANI-NFs) can be clearly observed from higher-magnification micrographs (inset Figure 5a), where aniline is polymerized to doped ES-based PANI in an acidic medium. They exhibit well-dispersed and interconnected ultrafine nanofibers with an average diameter of 17 nm (ChP-Figure 5a) and in the range of 5–10 nm (HT-Figure 5c), respectively, for both HT temperatures, not reported to date in the literature for pure PANI [22,77,80]. This linear structure of PANI has proved the enhancement of charge transport properties as reported later. However, Figure 5b,d,f exhibit the morphology of the PANI/MWCNT nanocomposites synthesized with an interesting in situ chemical polymerization process. They show the formation of highly porous networks without any agglomeration, as shown in their HRTEM micrographs [45,50]. This textural aspect clearly shows that functional groups served as active sites for nucleation during the in situ polymerization of aniline, resulting in more intimate contact between the MWCNT surface, which interacts with PANI-NFs via π bonds that might be beneficial for performance as supercapacitor electrodes. They offer an enormous number of open channels for rapid electrolyte ion transport and strongly facilitate the access of ions to the internal surface of these electroactive nanocomposites. Thus, O-MWCNTs were utilized to enhance their dispersion in aqueous media and strengthen their interfacial interaction with ultrafine PANI-NFs. The oxygen-containing functional groups (-COOH and -OH), identified through spectroscopy analysis, improve the hydrophilicity of the CNTs’ surface, thereby reducing agglomeration and promoting a more uniform distribution of PANI in the CNT matrix. This improved the interfacial bonding results and enhanced the electrochemical responses due to facilitated charge transfer at the PANI/MWCNT interface, as will be reported hereafter.

**Figure 6:**
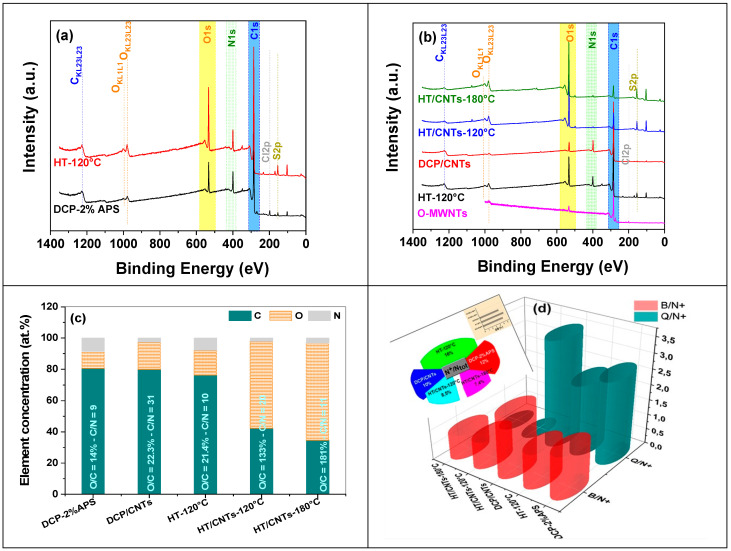
XPS survey of the synthesized PANI-NFs (**a**) and their nanocomposites (**b**). Quantitative estimation of their element concentrations (**c**) and protonation ratios (**d**).

To gain further information about the chemical composition and valence state of the produced PANI and PANI/O-MWCNT nanocomposites, depending on the growth conditions and O-MWCNT incorporation, we carried out XPS analysis. The wide survey spectra (Figure 6a,b) represent characteristic photoelectron peaks of these products, indicating the dominant elements: C1s (~284.6 eV), O1s (~532 eV), and N1s (~399 eV). Quantitative estimations of the element concentrations and protonation ratios for the PANI and PANI/O-MWCNT nanocomposites are shown in Figure 6c,d.

**Figure 7:**
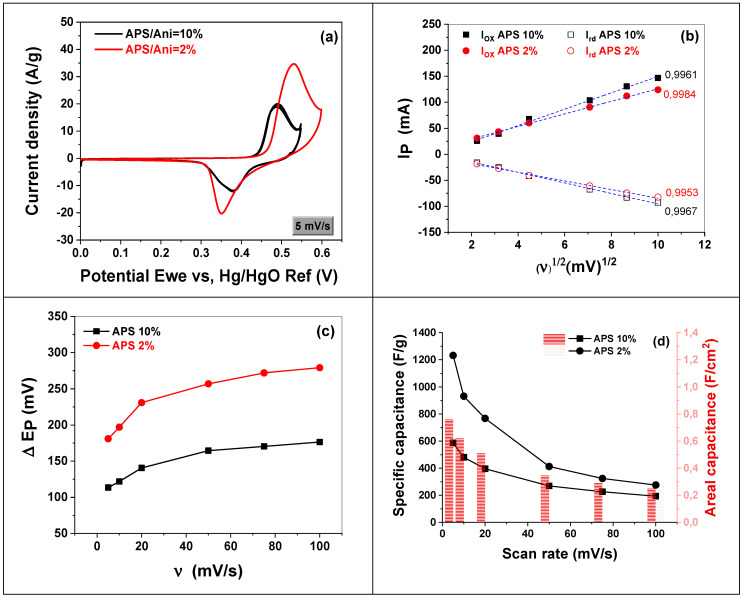
Effect of APS oxidant concentration as determined via CV tests at low scan rate (**a**), Ip vs. scan rate square (**b**), potential difference vs. scan rate (**c**), and specific and areal capacitance vs. scan rate (**d**).

The latter originates from the presence of PANI and provides a precise indication of the intrinsic oxidation and doping/protonation states inside the produced PANI and PANI/O-MWCNT nanocomposites [81,82,83]. In addition, some traces of the chloride ion peak (Cl1s around 198–200 eV) show that HCl added during the polymerization reaction not only provides an acidic medium but also dopes the final products [84,85,86,87]. The binding energies of the deconvoluted main peaks, as well as their relative area percentages, are reported in Figure 7 and Table S1 [82,83,84,85].

The high-resolution C1s spectrum of all the samples synthesized via the DCP and DCP and HT processes (Figure S1) can be deconvoluted into four main peaks centered at approximately 284.6, 286.3 ± 0.3, 287 ± 0.4, and 290 ± 1 eV, which are related to C–C/C=C aromatic rings (sp^2^C of the benzenoid ring labeled as “B”), C–N/C=N (quinonoid structure labeled as “Q”), C–N^+^/C=N^+^ (delocalized polaronic structures labeled as “B^+^”), and C=O/O–C=O/O–COO groups or π–π*, respectively [32,45,71,74,86,88,89]. The presence of C–O and C=O species in the range of 1.6 to 25 at.% in the C1s core-level spectra is indicative of the oxidation degree on PANI surfaces, reflecting the reactive nature of the most conjugated polymer surfaces or relating to the incorporation of oxygen-functionalized MWCNTs (in the case of the nanocomposite) [45,85]. The peaks associated with C–C/C=C (B rings) or C–H (284.6 eV) become predominant in all products, which decrease when incorporating O-MWCNTs (DCP process case) and increase again after the HT process step with increasing HT growth temperature. However, C–N/C=N (Q rings) peaks appear with O-MWCNT incorporation during DCP synthesis and decrease with increasing HT temperature. From these two characteristic peaks (B/Q ratio [85,86]), we can obtain an idea of the cross-linking degree inside the produced PANI-NFs. In addition, the C–N bond (~285.5–286 eV) is derived from cationic aniline units in the polymer chain [50,85,89,90]. The above-mentioned peaks are found to have different percentages with PANI only or after the HT step (Figure S1a_1_), or increase in the presence of O-MWCNTs (Figure S1a_2_); however, it can be clearly observed that the peak at 288.25 eV (C=O) disappears, is replaced by protonated rings (C–N^+^/C=N^+^) at 287.4 eV and appears again after adding the O-MWCNTs via the HT process at an increased temperature with a low concentration. Therefore, after the reinforcement of PANI-NFs with O-MWCNTs via the DCP method, the spectrum obtained in the nanocomposite case shows an additional dominant peak at 285.54 eV, which can be attributed to the appearance of C–N bonds due to the interaction of the PANI backbone with O-MWCNTs [51,91]. The percentage of C–N^+^/C=N^+^ groups increased, and C–O/C=O groups decreased after the HT step and with increasing HT growth temperature. We can also estimate that the defect density (C–O/C1s [92]) inside these PANI-NF products increases from 13.5% to 25.4% due to O-MWCNT incorporation and decreases to 8% or 15% after the HT process, respectively, in the experiments without or with O-MWCNTs (see Table S2). The high defect density originating from cross-linking leads to the most structurally stable product, resulting in lower conductivity than that for linear PANIs. The high-resolution O1s spectrum of these samples shows main peaks at 530.5 ± 0.1 and 532.49 ± 0.2 eV, owing to the presence of oxygen-containing functional groups inside the products corresponding to C–O and C=O, respectively (Table S2) [50,81,85]. The peaks’ assignments are very similar to those found in earlier studies by Kumar et al. [93] and Yue & Epstein [94]. Other oxygen molecule groups from adsorbed moisture/water in the PANI products were also detected at 534–535 eV [85]. These data also demonstrate that the produced ultrafine PANI is more oxygenated after the HT step and/or with oxygen-functionalized MWCNT incorporation, while the oxygen groups decrease upon increasing the HT process growth temperature. The N1s Lorentzian asymmetric spectrum of the PANI-NFs synthesized via the DCP process (Figure S1) can be deconvoluted into five or six different nitrogen-binding electronic states, signifying the presence of more than one kind of nitrogen inside these products (Table S3). The five main peaks found around 397.9 ± 0.2, 399.2 ± 0.3, 401, 402, and 403 eV are assigned to –N=/-N- (Q—neutral imine) [15,50,51,61,82,95,96]; -NH-/-NH_2_- (B—neutral amine) [44,50,61,74,81,82,96,97]; -NH^+^- (B^+^ polaron/protonated amine or nitrogen cationic radical) [32,44,61,81,82]; and =NH^+^- (Q+ bi-polaron/protonated imine) [32,82,95,96,98], with a lower one ascribed to oxide-protonated amine units (N^+^/ox). Hence, we can state that these N1s peaks are precisely deconvoluted into three distinct functional peaks, corresponding to the quinoid imine (Q) reduced state, the benzenoid amine (B), and positively charged nitrogen (N+)-oxidized and/or doped states depending on their atomic percentage, with the B-amine component located around 399.2 ± 0.3 eV still dominating [46,85]. This decreases slightly after the HT process step and increases upon incorporating O-MWCNTs. In addition, it is notable that the binding energies of the B main species in the produced PANI-NFs are shifted toward higher or lower binding energy values with respect to O-MWCNT incorporation, more so after the HT process. This shifting can be ascribed to electron transfer from PANI to O-MWCNTs in the nanohybrid configuration, which affects their subsequent properties, in good agreement with the Raman data shown in Figure 3c and in accordance with many other teams’ reported work [46,72,91,96]. In the case of PANI obtained after the HT process step, we found a slight increase in high-energy Q^+^ and B^+^ originating from the doping level in the polymer that favors a more doped ES, which is beneficial for electrical conductivity, as will be proved by further EC performance studies [45,74,85,90]. More precisely, the high percentage of cationic radical nitrogen (N^+^) indicates the doping of nitrogen protons in the products, thereby making them more conductive [99]. Thus, the protonation of PANI produces electronic defect states in the polymer chain related to the two charged nitrogen species as reported by Kumar et al.: polarons or bipolarons, which can be formed by the addition of protons to the neutral polymer chain [82,85,89,95].

To study a quantitative aspect of the electronic configuration resulting from intrinsic oxidation, the doping degree and defect density in the produced PANI-NFs were estimated and are presented in the form of a circle (inset of Figure 6d). This helps us determine the intrinsic oxidation level of PANI (via Q/B = imine/amine) and the doping degree or protonation level as the ratio between the positively charged nitrogen species and the total nitrogen (N^+^/N_tot_, B/N^+^, Q/N^+^). We can clearly observe the effect of adding the HT process step and the growth temperature, as well as the incorporation of O-MWCNTs, on these ratios. We suggest the successful polymerization of aniline with area fractions of 80–85 at.%, which gives the protonation extent in PANI. Likewise, according to the C1s and O1s spectra, the calculated C/O ratios decrease upon adding the HT process step and significantly decrease upon the incorporation of O-MWCNTs (Figure 6c). However, in the case of these PANI ultrafine nanofiber products, the ratio of total C1s to N1s (C/N) is in a range [100] that almost corresponds to the theoretical value of the formula (C_6_H_5_N)n (C/N = 6). Moreover, the total proportion of the quinoid amine group (=N-) and positive nitrogen cationic radical (N^+^) contribution (Q/Q^+^) could be up to 50%, which indicates the high electrical conductivity of the PANI-NF products as previously reported by Liu’s team [74]. However, the ratio of (B/(B^+^Q^+^)) is found to be between 0.9 and 1, suggesting that these PANI-NFs are oxidized ES, favoring various intrinsic redox states of PANI as will be proved via their electrochemical results [26,32]. We can observe that the presence of O-MWCNTs, with and without the HT process, remarkably increases the protonated N^+^ either in polaron or bipolaron form (Figure 6d), confirming the effective doping of PANI-NFs in the ES state. Generally, well-doped PANI exhibited an almost total absence of a neutral imine peak in our investigation (Table S3). Consequently, the above XPS surface characterization clearly demonstrates that the PANI ultrafine nanofibers in the heterostructures, owing to doping and oxidation levels, are in the form of half-oxidized ES, which is in accordance with the FTIR and Raman analysis results. Hence, they can achieve their highly conductive state either through the protonation of the imine nitrogen (-N= or Q^+^) in its ES or through the oxidation of the amine nitrogen (–NH– or B^+^), depending on the polymerization condition (after the HT process and/or by incorporating O-MWCNTs).

### 3.2. Electrochemical Properties of PANI-NF and the PANI/O-MWCNT Nanocomposites

The capacitive EC performance of pure PANI and PANI/O-MWCNT nanocomposite-based electrodes was investigated and evaluated via their electrochemical and transport properties by using three main tests—CV, GCD, and EIS—as mentioned in Section IV. The tests were performed in a three-electrode cell setup using an aqueous electrolyte (6M KOH) to identify their potential future application, especially in energy storage and biosensing devices. First, all the products were found to exhibit the characteristic CV curves of Faradaic or redox (pseudocapacitive) behavior [6,101], which originate from the doping state changes of PANI with some shifts depending on the added quantity of APS, growth synthesis method (DCP or HT), and incorporation of O-MWCNTs via in situ chemical polymerization. The CV results generally present similar profile curves, deviating from the ideal rectangular shape corresponding to electric double-layer capacitance (ELDC) [6,10], with one dominant pair of redox characteristic peaks, implying that the PANI ultrafine nanofibers have low internal resistance with a fast selective redox rate (Figure S2). It is noteworthy that, as is generally found with high-performance EC measurements, the CV curves show that the anodic peaks (oxidation) of the electrodes shift positively, while their cathodic peaks (reduction) shift negatively when increasing the scan rate (5 to 100 mV·s^−1^) with a slight change, implying good electrochemical stability. These results indicate that the fast redox reactions occur at the electrolyte/electrode interface, which is mainly due to the internal resistance of the electrodes, and reveal higher charge storage. In addition, the shifting of the redox peaks with a ΔV deviation of less than 200 mV indicates excellent electrochemical reversibility and outstanding high-rate performance, as also proved by Laviron’s work [102].

#### 3.2.1. APS Effect

To study the influence of the APS/Ani molarity ratio in first-step chemical synthesis (DCP) on these products’ electrochemical behavior, the CV curves illustrated in Figure 7 were generated at a low scan rate (5 mV·s^−1^). These curves demonstrate several broad redox peaks, revealing good Faradaic redox behavior and good EC process reversibility (Figure 7a) [17,22]. They clearly show the main redox peaks around 0.49–0.52 V and 0.35–0.39 V at low scan rates (5–20 mV·s^−1^), which can be ascribed to the comprehensive effect of the homogeneous structural changes in the PANI oxidation state, as initially proved in the XPS results [22,44]. There is a possible selective redox transition between a leucoemeraldine state (semiconducting or insulating state) and a polaronic ES (conducting state) of these PANI-NFs due to the doping state, as studied previously [17,21,45,103,104,105]. Obviously, a lower APS quantity (APS/Ani = 2%) resulted in a much higher redox current and a shift to low/high potential (0.39/0.49 to 0.35/0.52 V), suggesting high conductivity and low internal resistance of the produced PANI-NFs, probably confirming higher electron transport due to their electronic state via their protonation, as proved via FTIR and XPS spectroscopic analyses.

It is also noted that with increasing scan rate, the CV curves have a similar shape at two different APS/ANI molarity ratios, with slight distortion at a high scan rate (Figure S2), implying a reversible surface-controlled redox process in the diffusive behavior of the produced PANI-NFs (Figure 7b,c). Therefore, we found that the APS oxidant agent concentration plays a more significant role in the specific capacitance of PANI nanofibers, as reported previously in T. Li’s work [106]. It is established to be around 1200 F/g (equivalent to 1.2 F/cm^2^) with a lower amount of APS (twice as high as that for high APS content), which is favored by rich benzenoid units with a high degree of electron delocalization in the structure, as reported in the FTIR and Raman results. It is clearly much higher than the reported values in the literature. Using the relationship between the specific gravimetric capacity and incorporated mass

C_a_ = C_s_ × m_a_ (4)

where m_a_ = m/A = active mass per surface area (g/cm^2^), and based on the mass loading, the corresponding areal capacity is estimated to be in the range of 0.25–1.23 F/cm^2^ (Figure 7d).

#### 3.2.2. Growth Method Effect

**Figure 8:**
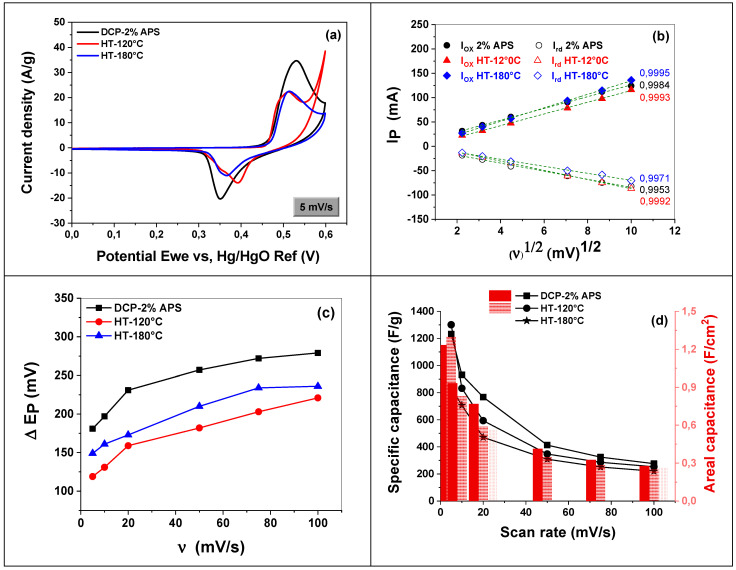
PANI-NF CV tests before and after the HP process at two different temperatures (**a**), their current responses (**b**), their potential differences (**c**), and their specific and areal capacitances (**d**).

**Figure 9:**
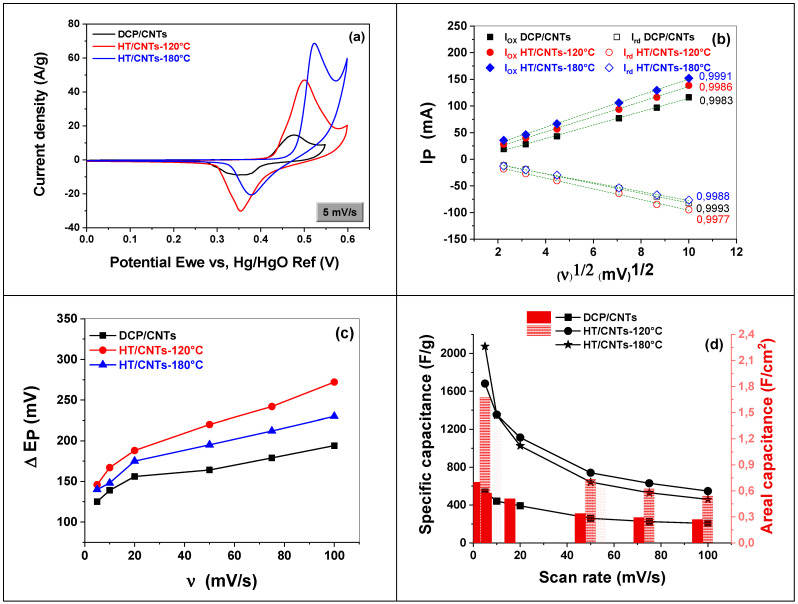
CV tests of PANI-NFs with and without O-MWCNTs before and after the HP process at two different temperatures (**a**), their current responses (**b**), their potential differences (**c**), and their specific and areal capacitances (**d**).

For pure PANI after the HT process at the optimized temperature of 120 °C, it can be observed that the selective and homogeneous CV curves have a larger area under the redox curve, which indicates higher capacitance (Figure 8) in comparison with PANI obtained at a high growth temperature (180 °C). However, the similar CV curves illustrated in Figure 9 show the opposite phenomena when incorporating O-MWCNTs into the PANI matrix using the in situ chemical polymerization process, where the enclosed area of the CV plots decreases, and becomes larger at high HT growth temperatures. These effects must be due to the creation of more active sites for redox kinetics, such as the doping of protonated nitrogen atoms inside the PANI-NF chain skeleton, as well as good contact with O-MWCNTs. Thus, the idea highlighted in this work is that the APS oxidant agent dispersed with O-MWCNTs during the ultrasonication step before the growth process allows very good integration of PANI-NFs into the network of O-MWCNTs via in situ polymerization. This allows for their effectiveness through enhanced conductivity. The corresponding areal capacitances were estimated to be in the ranges of 0.25–1.3 F/cm^2^ (Figure 8d) and 0.26–2.07 F/cm^2^ (Figure 9d).

In addition, excellent penetration of the KOH electrolyte into the nanocomposites arose from both the nanoscale size of the ultrafine PANI in nanofiber form (mean diameter between 5 and 17 nm) and hence their electronic state, as proved by XPS analysis via the N^+^/N_tot_ ratio (inset Figure 6d), as well as from their network configuration, with highly porous functionalized MWCNTs for ion adsorption, as also described by Jasna [107] and Guo’s teams [108]. Consequently, the selective redox peak became more evident in these two nanocomposites, which originates from the strong redox transitions in these doped and functionalized PANI-NFs. The major peak currents I_pa_ and I_pc_ from these cyclic voltammograms are plotted against the square root of scan rate (Randles Sevcik plot) (Figure 7b, Figure 8b and Figure 9b), and their redox peak potential separations are plotted against the scan rate (Figure 7c, Figure 8c and Figure 9c). A linear and increasing relationship between I_pa_ and I_pc_ can be observed, demonstrating a good rate capability and the occurrence of diffusion-controlled ionic species transport kinetics at the interface between the electrode and the basic electrolyte. Hence, they confirm that the energy storage kinetics originate from the surface-controlled redox process of PANI-NF and PANI-NF/O-MWCNT nanocomposites in the bulk [35,106,107]. However, the relationship between ΔE_p_ and ν indicates that these electroactive products exhibit an excellent quasi-reversible redox reaction [22].

The PANI-NF/MWCNT nanocomposite obtained at 180 °C over 6 h clearly exhibits a larger enclosed area of the CV plot than the other two nanocomposites at the same scan rate (5 mV·s^−1^). This indicates higher electrochemical reactivity with an increase in the charge storage capacity compared with that at the lowest temperature or before adding the HT process step. This suggests a fast diffusion rate within the redox phase affected by HT conditions, especially at higher temperatures, contrary to the case of PANI, where capacitive performance is also limited due to a serious agglomeration phenomenon reducing the effective active surface area. To estimate this phenomenon, the diffusion coefficients were calculated for all the products using the modified Randle–Sevcik equation for quasi-reversible systems, as follows [37,108]:

(5) $$I_{p}^{quasi}=2.65\times 10^{5}ACn^{3/2}D^{1/2}v^{1/2}$$

where $I_{p}^{quasi}$ is the peak current, *A* is the electrode area in cm^2^, *C* is the concentration of the KOH electrolyte in mol/cm^3^, *n* is the number of electrons participating in the redox reaction (*n* = 1), *D* is the diffusion coefficient in cm^2^/s, and ν is the scan rate in V/s. The best obtained *D* values at 5 mV/s were around 3.73 × 10^−7^ and 1.75 × 10^−7^ cm^2^/s for PANI-NFs/O-MWCNTs (HT-180 °C and HT-120 °C, respectively), in comparison with PANI-NFs, which had values of around 0.39 × 10^−7^ and 0.96 × 10^−7^ cm^2^/s (after the HT and DCP processes, respectively), defining the rate of the electrochemical reaction and the obtained capacitance values.

**Figure 10:**
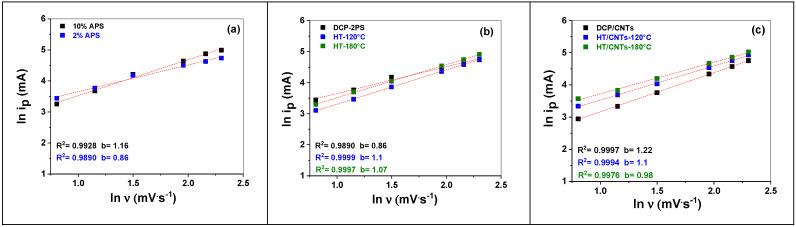
Logarithm of current (i_p_) vs. logarithm of scan rate plots of all samples depending on APS content (**a**), synthesis method (**b**), and O-MWCNT incorporation (**c**).

Referring to the work of Xiang Chu et al. [9], the Faradaic mechanism can be classified into three kinds: underpotential deposition, redox pseudocapacitance, and intercalation pseudocapacitance. The last one focuses on storage mechanisms and kinetics, which are widely used to fabricate pseudocapacitance supercapacitors based on Faradaic and non-Faradaic behavior. The difference between these two behaviors is mainly determined through analyzing the features of current measurements from CV curves at different scan rates by using the following power-law relationship:

i = aν^b^ (6)

where i is the current measured at a fixed potential, ν is the scan rate, and a and b are two adjustable parameters derived from the fitted linear curve that provide information about the electrochemical reactions. Moreover, the b values can be estimated as the slope of the plot ln(i) versus ln(υ) (Figure 10). In general, there are two important considerations for b values: b = 0.5 corresponds to the diffusion-controlled Faradaic intercalation process that occurs in the bulk (battery behavior), and b = 1 is attributed to the surface-controlled process (capacitive behavior) [4,10,51,70,105,109]. In this investigation, it is concluded that the b values of the samples are mostly between 0.5 and 1, which signifies a “transition” area between pseudocapacitive and battery-type behavior, though a clear boundary is not easy to define, and two behaviors are possible. Generally, we suppose that the smaller the b value, the larger the contribution from diffusion-controlled intercalation processes, while the capacitive contribution increases with increasing b value. We found b values of 0.86 and 1.1 for PANI after the DCP and HT processes, respectively; however, they were 1.2, 1.1, and 0.98 for PANI with O-MWCNT incorporation after DCP, HT-120 °C, and HT-180 °C, respectively.

These results indicate that the charge storage kinetics of these PANI-NFs and their nanocomposites are due to a combined and interplaying effect of the capacitive and diffusion-controlled mechanisms (providing a clear indication of the surface redox and ion exchange during the redox reaction), as found previously in various studies [4,9,70,110,111].

**Figure 11:**
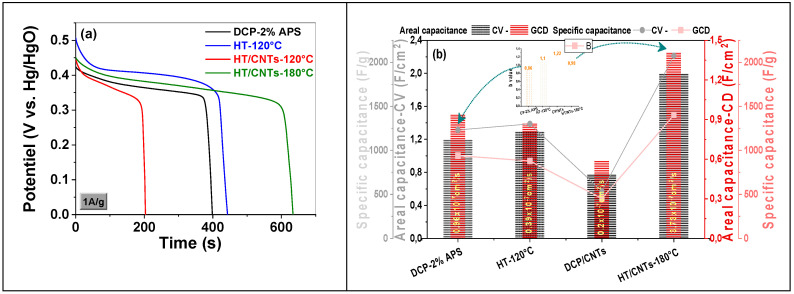
Discharge curves from galvanostatic CD tests of the best PANI-NF samples with and without O-MWCNTs before and after the HT process (**a**), their areal capacitance from CV or areal capacitance from GCD, and their diffusion coefficient (**b**).

To reinforce the EC performance of synthesized PANI-NF products, GCD tests were performed at various current densities (from 1 to 100 A·g^−1^), and the discharge curves of the best products at low current density are illustrated in Figure 11. The nonlinear curves with a near-plateau are consistent with the abovementioned CV mechanism results and confirm the Faradaic behavior of the PANI-based ultrafine nanofibers. The apparent contradiction between the kinetic analysis and the GCD characteristics must be taken into account: (i) in pseudocapacitive systems, such as PANI-based electrodes, fast and reversible surface or near-surface Faradaic reactions can occur at well-defined redox potentials, giving rise to quasi-plateau features in the GCD curves; (ii) the b value highlights the kinetic origin of the charge storage and indicates that the current scales almost linearly with the scan rate (b ≈ 1), confirming that the dominant process is surface-controlled rather than diffusion-limited. In conclusion, in the case of supercapacitors having the PANI/O-MWCNT composite as the active electrode material, the thin PANI layer and the highly conductive MWCNT network allow for fast electron transport and short ion-diffusion paths, allowing surface redox reactions to dominate, while exhibiting redox-related plateaus. The effects of the APS/Ani molarity ratio, synthesis method, and HT growth temperature on the discharge duration are clearly revealed. The PANI-NF/MWCNT nanocomposite prepared under 6 h/180 °C HT conditions exhibited a longer discharge time (635 s) compared with other as-synthesized products at the same current density. This is because the internal resistance of the nanocomposite is reduced when O-MWCNTs are incorporated into the PANI matrix, hence improving the e-transfer and diffusion. It significantly enhances the effective energy storage estimated capacity of the electroactive synthesized ultrafine PANI nanofibers, as reported below [35,97].

**Scheme 1:**
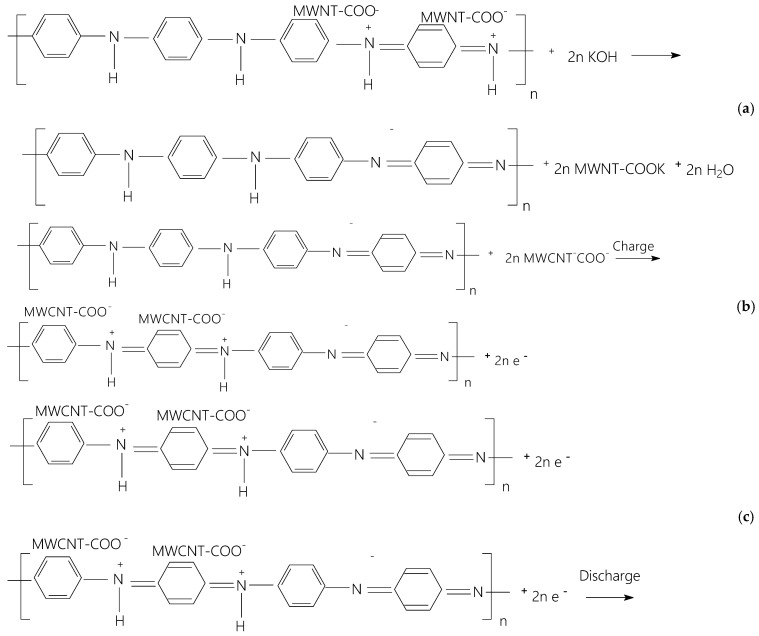
(**a**) Chemical interaction of PANI doped with MWCNT–COO^−^ ions with KOH, (**b**) charge process, and (**c**) discharge process.

The estimated areal/specific capacitance values of the best as-synthesized PANI ultrafine nanofibers obtained from the CV/CD curves are illustrated in Figure 8d, Figure 9d and Figure 11b. These curves clearly show that areal capacitance decreases with increasing scan rates or specific capacitance current densities (Figure S2), showing the effect of contact time during electrochemical measurements. Specifically, exceptionally high areal (specific) capacitances of 2074 F·cm^−2^ (2821 F·g^−1^), 1301 F·cm^−2^ (1751 F·g^−1^), and 1232 F·cm^−2^ (1879 F·g^−1^) were obtained at a low scan rate (5 mV·s^−1^) and current density (1 A·g^−1^) for the best PANI-NFs and PANI-NF/MWCNT nanocomposites prepared under optimized conditions via either a chemical or hydrothermal process. They reflect intrinsic electrochemical activity rather than practical device performance. The results from the EC analysis are in accordance with the doping level, as well as the presence of positively charged amines –NH^+^ (B^+^) and NH^+^_2_, with the existence of polarons/bipolarons. As reported previously, =N– (Q) and –NH– (B) play key roles in improving the specific capacitance of N-doped PANI due to their pseudocapacitive contribution, while –NH^+^ can enhance the conductivity of the materials and therefore increase the capacitance [15]. These capacitance values of supercapacitors must take into account a mechanism in which (a) carbon nanotubes, which present a high surface area, high conductivity, and considerable mechanical stability, play a significant role in preventing PANI decline in charge/discharge processes by ensuring fast electron transport; (b) PANI-ES, which corresponds to PANI doped with MWCNT-COO^−^ ions, leads to MWCNT-COOK and a polyaniline–emeraldine base in the presence of KOH; and (c) there is a nanofibrous structure that ensures fast access of ions. The highest capacitance values can be explained only if we accept that the mechanism is as shown in Scheme 1.

However, to investigate how the degradation mechanism is mitigated under current conditions, we plan to perform charge/discharge studies using active material composites as electrodes, with varying concentrations of MWCNT-O in the mass of PANI fibers and fibers with various diameters. Additionally, in situ and ex situ Raman studies will be performed in future work.

**Figure 12:**
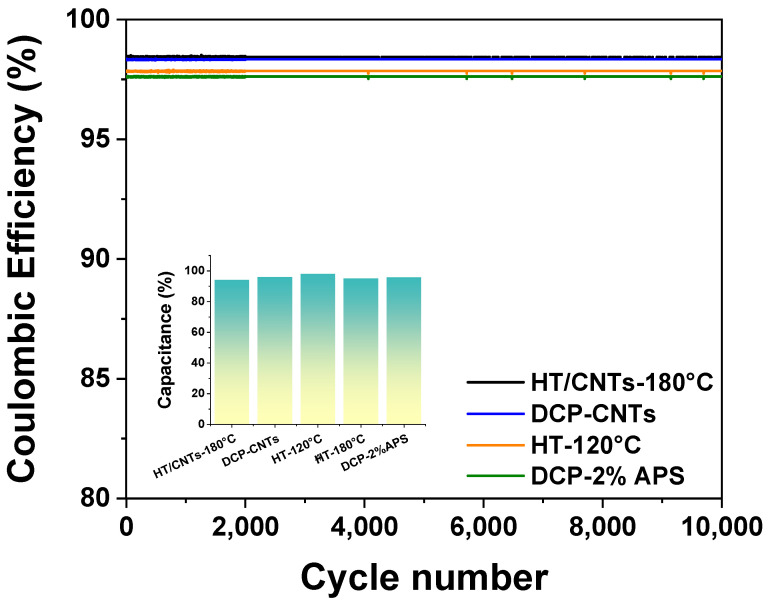
Coulombic efficiency (capacitance retention) as a function of cycle number at a current density of 30 A·g^−1^ for the best PANI ultrafine nanofibers and their nanocomposites with O-MWCNTs synthesized with DCP or after the HT process.

Evaluating cyclic stability has a critical role in assessing the long-term durability and viability of these synthesized electroactive materials for use in supercapacitor-based energy storage applications. The cyclic stability of these synthesized compounds is shown in Figure 12.

According to Figure 12, the electrode retained >94% of its initial capacitance after 10,000 continuous charge–discharge cycles at 30 A/g, demonstrating outstanding cycling stability and high rate capability. This durability, supported by the almost preserved structural features in the post-cycling FTIR spectra shown in Figure S3, confirms strong and robust resistance to KOH electrolyte degradation and structural integrity over prolonged high-current operation.

This remarkable retention can be attributed to the robust architecture of the as-synthesized ultrafine nanofiber PANI/O-MWCNT nanocomposite, which provides mechanical integrity against volume changes during redox processes. In addition, the strong interfacial interaction between PANI nanofibers and functionalized CNTs facilitates efficient charge transfer kinetics, while the intimate contact with the NiF current collector ensures good electrical conductivity and mechanical adhesion under electrochemical operating conditions. In recent years, numerous studies have explored PANI and PANI-based composites in alkaline electrolytes, such as KOH and NaOH, often showing favorable capacitive responses compared with acidic media [37,112,113,114]. Polyaniline (PANI) has demonstrated significant electrochemical activity in alkaline electrolytes like KOH, maintaining good redox reversibility and capacitive performance even without the strong protonation typical of acidic environments. The charge storage mechanism relies on reversible transitions between leucoemeraldine, emeraldine, and pernigraniline states, driven by OH^−^ ion insertion/extraction during cycling. The high ionic conductivity and small ionic radius of K^+^ (3.31 Å) further enhance the charge transfer kinetics through the ultrafine nanofiber morphology, hence contributing to higher specific capacitance. The high specific capacitance and electrochemical stability of supercapacitors based on PANI–emeraldine salt and O-MWCNTs are the result of the synergistic effect between O-MWCNTs and PANI, where O-MWCNTs, which mechanically stabilize the PANI chain, create micro-environments favorable for partial protonation, as a consequence of the compensation of the positive charges on the PANI macromolecular chain by the carboxyl groups (-COOH); this improves electrical conductivity and prevents the structural degradation of PANI in an alkaline environment. In the case of supercapacitors having only PANI-ES as the active electrode material in the presence of the electrolyte (6M KOH), deprotonation takes place, resulting in PANI-EB, with a decrease in conductivity, repeated swelling/contraction, and cracking of the electrode. In contrast with this behavior, in the case of the PANI-NF/O-MWCNTs, we must consider the interfacial confinement and buffering effect as the main mechanism underlying the high retention rate. In this context, we must note that (a) PANI is deposited as a thin layer on MWCNTs, with the macromolecular chains being anchored on the surface of the carbon nanotubes, which prevents large volumetric changes, keeps redox reactions on the surface, and minimizes structural degradation, and (b) the –COOH groups function as a local acid buffer, maintaining the partial protonation of PANI, thus contributing to slowing the irreversible transformation of PANI-ES into PANI-EB, so that even in the presence of the 6 M KOH electrolyte, PANI remains electrochemically active, with capacitance loss occurring slowly. All of this allows highly reversible pseudocapacitive behavior over prolonged cycling.

For better comparison of the as-synthesized PANI-NF products, the specific capacitance values reported by several recent studies using different electrolytes are listed in Table 2. The use of the potassium hydroxide (KOH) electrolyte in our study was motivated by several advantageous EC properties. Aqueous KOH offers high ionic conductivity and a broad EC stability window, which significantly contribute to enhancing the EC performance and cycling stability of PANI-NF electrodes. In particular, the alkaline environment facilitated by KOH improves the accessibility and transport of electrolyte ions, which synergize with the oxygen-functionalized MWCNTs to boost the overall electrode performance, as demonstrated by the results obtained in comparison with those reported to date in the literature. More precisely, in this alkaline electrolyte, hydroxide ions (OH^−^) actively participate in the redox reactions inside PANI-NF-based products between their different oxidation states by interacting with the polyaniline backbone, facilitating the doping and dedoping processes [79,114]. This interaction promotes rapid Faradaic charge transfer processes, resulting in fast and reversible charge storage. Additionally, the structural and morphological features of the PANI nanofibers combined with O-MWCNTs provide a large surface area and enhanced electrical conductivity, which further support the efficient double-layer and pseudocapacitive mechanisms discussed above. Consequently, the PANI/KOH system exhibits charge storage through surface redox reactions and electrochemical double-layer capacitance, resulting in a higher specific capacitance when PANI is combined with MWCNTs in nanocomposites for supercapacitor electrode applications, as demonstrated in this study. However, a comparative analysis reveals that most electrochemical measurements on PANI nanocomposites have been conducted using acidic electrolytes, such as H_2_SO_4_ and KCl, which may lead to corrosion and pose environmental safety risks over time.

**Figure 13:**
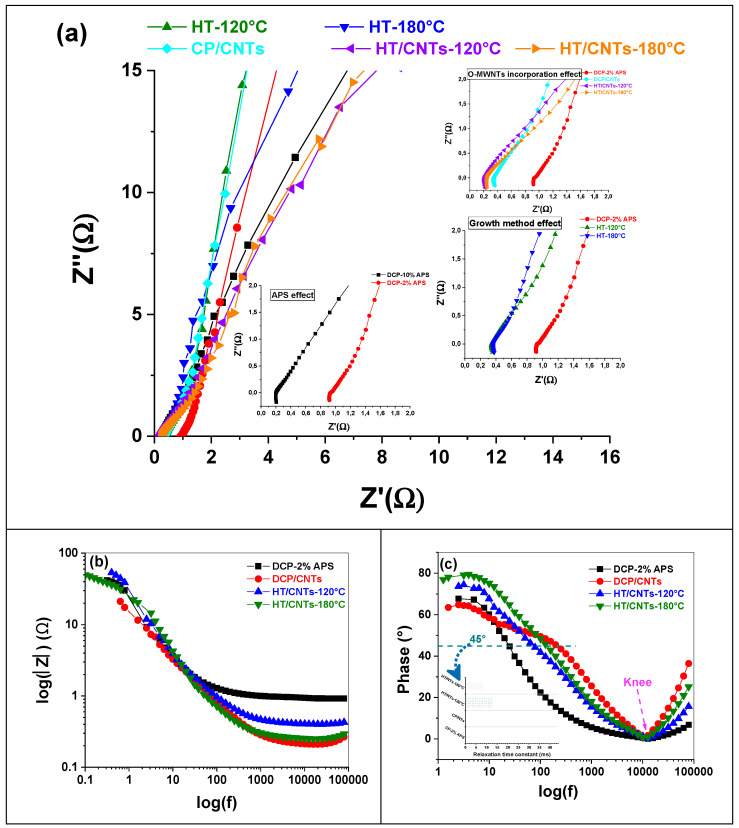
(**a**) Nyquist plots of as-prepared PANI-NFs with and without O-MWCNT incorporation (inset: high-frequency region), and (**b**,**c**) their Bode plots (inset: deduced relaxation time constant).

Moreover, for further electrochemical information on pure PANI-NFs and their nanocomposites after O-MWCNT incorporation for the subsequent generation of supercapacitor devices, the impedance spectra were measured in the frequency range from 0.01 to 10^5^ Hz at open circuit potential (E_oc_) (Figure 13a). Almost all the Nyquist plots originate from two parts: the partial semicircle (abscissa real *X*-axis, Z’) in the high-frequency region due to the dispersion effect, and a straight deviated line from the imaginary axis (ordinate *Y*-axis, Z″) at low frequency [124], revealing their electronic conductivity during the redox process in solution through charge transfer characteristics at solid–liquid and solid–solid interfaces [12,125,126]. We found that all these Nyquist plots exhibited a smaller semicircle, indicating lower interfacial charge transfer resistance between 0.1 and 0.2 Ω, which means a faster electron transfer process corresponding to the Faradaic reaction on the surface, as demonstrated previously [35,127]. Meanwhile, it should be noted that all the products also show less deviation (less than 45°) of the sloping line, indicating shorter diffusion resistance and more than quasi-ideal pseudocapacitor (Faradaic) behavior. More precisely, we illustrate that the internal resistance, where the semicircle intersects the real axis—consisting of the equivalent series resistance (ESR) of the ionic resistance of the electrolyte, the intrinsic resistance of the active PANI-NF products, and the contact resistance at the electroactive material/current collector interface—was found to be affected by the synthesis method (0.9 and 0.4 Ω with the DCP and HT process, respectively) and by the incorporation of O-MWCNTs (0.9 and 0.2–0.4 Ω, without and with O-MWCNTs, respectively).

Hence, they prove shorter ion diffusion and a lower ionic flow resistance path length in these electroactive PANI ultrafine nanofibers and/or upon the incorporation of oxygen-functionalized MWCNTs. This probably originates from the PANI sample texture supporting linearity at the nanoscale (around 5 to 10 nm), as well as from the specific contact surface providing new electroactive sites in the presence of O-MWCNTs, enhancing the electrochemical surface area. Consequently, efficient access of electrolyte ions to the surface of electroactive materials, which causes a high specific capacitance confirmed by the above-obtained values, is achieved. Figure 13b depicts a Bode plot of the best products depending on the synthesis method and the incorporation of O-MWCNTs. It contains the data plotted in the impedance modulus and in phase-versus-frequency format. The impedance in the KOH electrolyte Bode plot decreases with increasing frequency, whereas the phase angle decreases with increasing frequency until the knee frequency, and then increases rapidly with further frequency increase [126]. For the current work, the knee frequency of these produced PANI-NFs and their nanocomposites with O-MWCNTs is around 10 kHz, and the phase angle is found to be between 60° and 80° at low frequencies up to 1 Hz, suggesting the ideal capacitor behavior of the nanocomposite. The significance of the so-called knee frequency in the Bode plot is that it is the critical frequency where all surface area is accessed, i.e., saturated, as reported by Tayel’s work [128]. Furthermore, the relaxation time constant (τ_0_) for these products was calculated from the equation τ0=1f0, where *f*_0_ possesses a phase angle of 45°, at which point the capacitive and resistive impedances are equal [35,129,130]. The τ_0_ was found to be affected by the incorporation of O-MWCNTs (via DCP), being eight times lower; however, with the introduction of the HT step, the relaxation time was found to be 13 and 8 ms, corresponding to a response frequency of 77 and 125 Hz depending on the HT growth temperature at 120 and 180 °C, respectively (see inset of Figure 13c). It is obvious that a higher knee frequency corresponds to a better rate capability, resulting in a decreased relaxation time, which surpasses the results found in the investigations of Yang [52] and Shao [129], indicating faster ion diffusion and more efficient charge transfer in the electrode material.

## 4. Conclusions

In this work, pure PANI-NFs and their MWCNT nanocomposites functionalized with functional groups containing oxygen (such as carboxylic groups) were successfully synthesized using two simple and low-cost dilution chemical polymerization (DCP) and hydrothermal (HT) processes. We demonstrate that the precursor ratio, the simultaneous doping–polymerization method, and the growth hydrothermal temperature play a vital role in determining the physical, chemical, and electrochemical properties. Using XPS, FTIR spectroscopy, Raman scattering, TG, and SEM, it was demonstrated that (i) ultrafine nanofibers with an average diameter in the range of 8–17 nm were obtained; (ii) according to XRD studies, the crystallinity of the PANI/O-MWCNT nanocomposites is similar to that of PANI; (iii) the two constituents strongly interacted, which facilitated electron transfer at the interface of the PANI and O-MWCNT layers, according to the FTIR spectroscopy and Raman scattering results; (iv) TG-DTA studies proved that PANI/O-MWCNT nanocomposites showed good stability up to 640–680 °C, in contrast with pure PANI; and (v) the XPS studies demonstrated that, upon reinforcement of PANI with O-MWCNTs via the DCP method, an additional dominant peak at 285.54 eV is reported, which is assigned to the new C–N bonds generated by the interaction of the PANI backbone with O-MWCNTs. Concerning the use of PANI/O-MWNT composites as active electrode materials in electrochemical supercapacitors, a higher specific capacitance of 1410 F/g^−1^ (2074 F/g) at a current density of 1 A·s^−1^ (scan rate of 5 mV·s^−1^) in aqueous alkaline electrolyte was reported under low mass loading and favorable laboratory conditions as superior to that reported in the literature. These results clearly indicate that the post-synthesis HT process is an easy and highly efficient way to promote the formation of PANI nanofibers (8 nm) without any additives or templates, further improving their EC performance as electroactive materials with good rate capability and lower internal R_0_/interfacial charge transfer R_Փ_ resistance of around 0.2 Ω. These electrochemical properties position these ultrafine materials as promising candidates for flexible supercapacitor applications. The development and evaluation of two-electrode full devices, crucial for assessing real-world performance, will be the focus of future work.

## Figures and Tables

**Table 1:** Raman features of synthesized PANI/O-MWCNT nanocomposites.

Samples	D (cm^−1^)	G (cm^−1^)	I_D_/I_G_
O-MWCNTs	1332	1592	1.07
PANI/O-MWCNTs (DCP)	1326	1589	0,53
**PANI/O-MWCNTs (HT-120 °C)**	1345	1588	0.78
**PANI/O-MWCNTs (HT-180 °C)**	1350	1572	0.88

**Table 2:** EC performance comparison of PANI combined with different materials.

Electroactive Material	Electrolyte	Specific Capacitance (F·g^−1^)	Reference
PANI-ultrafine NFs PANI-ultrafine NFs/O-MWCNTs	6M KOH	2074 (2.07 F/cm^2^)-CV 1410 (1.41 F/cm^2^)-GCD	This work
PANI/RGO/MWCNTs	1M H_2_SO_4_	498	[115]
Nano-PANI/HCS	1M H_2_SO_4_	435	[116]
PANI/MWCNT	1M H_2_SO_4_	663	[108]
GO/PANI SG/PANI	1M HCl	1095 478	[117]
PANI/MWCNT	1M H_2_SO_4_	360	[118]
NiO/PANI-MWCNT	10 mL KCl	356.54	[36]
rGO/PANI	1M H_2_SO_4_	524.4	[119]
CNTs/PANI	1M H_2_SO_4_	406	[120]
PANI	1M H_2_SO_4_	857.2	[106]
PANI/MWCNT	1M H_2_SO_4_	1551	[35]
MSG/PANI	1M H_2_SO_4_	912	[121]
PANI:PSS/Fe-FLG	0.1M HCl 0.1M KCL	768.6 659.2	[32]
RGO/PANI NTs	1M KCl 1M H_2_SO_4_	876.43 1081.03	[122]
GSA/PANI HS	1M H_2_SO_4_	546	[33]
Porous G/PANI nanoarrays	1M H_2_SO_4_	752	[44]
GH/SWCNTs/PANI	1M H_2_SO_4_	145	[59]
CuO@NiO/PANI/MWCNT	3M NaOH	1372	[37]
PANI/ND@GN	1M KOH	150.20	[100]
PANI/rGO-HT	1M H_2_SO_4_	420	[30]
LDH/PANI	(0.5 mol/L) K_3_[Fe(CN)_6_]	592.5	[28]
PANI	1M H_2_SO_4_	387.7	[7]
PANI-MWCNTs-Ni(OH)_2_	1M KOH	1917	[112]
PANI/CNF	1M H_2_SO_4_	493.75	[17]
PANI-g-CF	1M H_2_SO_4_	1178	[123]
PANI doped with HSO_4_^−^ or Cl^−^ ions	1M KOH	155.75 and 167.75	[114]
PANI nanofibers	H_3_PO_4_/PVA	280.4	[79]

## Data Availability

The original contributions presented in this study are included in the article/Supplementary Material. Further inquiries can be directed to the corresponding authors.

## Supplementary Materials

The following supporting information can be downloaded at: https://www.mdpi.com/article/10.3390/ma19071356/s1, Figure S1: The deconvolution of the XPS C1S, N1s, and O1s spectra of pure PANI-NFs (a1,b1,c1) and their nanocom-posites with O-MWCNTs (a2,b2,c2), Figure S2: CV and GCD curves at different scan rates and current densities, Table S1: The deconvolution of the XPS C1s spectra, Table S2: The deconvolution of the XPS O1s spectra, Table S3: The deconvolution of the XPS N1s spectra, Figure S3: Structural stability of PANI-NFs after electrochemical cycling: FTIR spectra of doping retention and deprotonation effects. References [131,132,133,134] are cited in the Supplementary Materials.
