# Supplementary material for: Exploring the Synergistic Effects of Ultrafine Polyaniline Nanofibers and Oxygen-Modified Multi-Walled Carbon Nanotubes on Enhancing Pseudocapacitive Electrochemical Performance for Advanced Supercapacitors
Source: Materials (Basel). 2026 Mar 29;19(7):1356. doi: 10.3390/ma19071356 (PMC13074056; doi:10.3390/ma19071356)
Supplement: Supplementary file 1 [file materials-19-01356-s001.zip › materials-4157049-supplementary.pdf]

materials

MDPI

Exploring the Synergistic Effects of Ultrafine Polyaniline Nanofibers and Oxygen-Modified Multi-Walled Carbon Nanotubes on Enhancing Pseudocapacitive Electrochemical Performance for Advanced Supercapacitors

Fahima Djefaflia [1,2,*], Ouanassa Guellati [1,2,*], Assia Nait Merzoug [1,2], Aicha Harat [1], Jamal El Haskouri [3], Izabela Janowska [4] and Mihaela Baibarac [5,*]

1 Laboratoire d’Etude et de Recherche des Etats Condensés (LEREC), Physic Department, University of Annaba, BP. 12, Annaba 23000, Algeria; abenlala@yahoo.fr (A.N.M.); harat_aicha@yahoo.fr (A.H.)
2 Faculty of Science and Technology, University of Souk Ahras, BP. 1553, Souk-Ahras 41000, Algeria
3 Instituto de Ciencias de los Materiales, Universitat de València, C/Catedrático José Beltrán, 2, 46980 Paterna, Valencia, Spain; jamal.haskouri@uv.es
4 Institut de Chimie et Procédés Pour l’Énergie, l’Environnement et la Santé (ICPEES), CNRS UMR 7515, Université de Strasbourg, 25 Rue Becquerel, 67087 Strasbourg, France; janowskai@unistra.fr
5 National Institute of Materials Physics, Atomistilor Street, No 405 A, 077125 Magurele, Romania
* Correspondence: f.djefaflia@univ-soukahras.dz (F.D.); o.gualati@univ-soukahras.dz (O.G.); barac@infim.ro (M.B.)

Supplementary Material

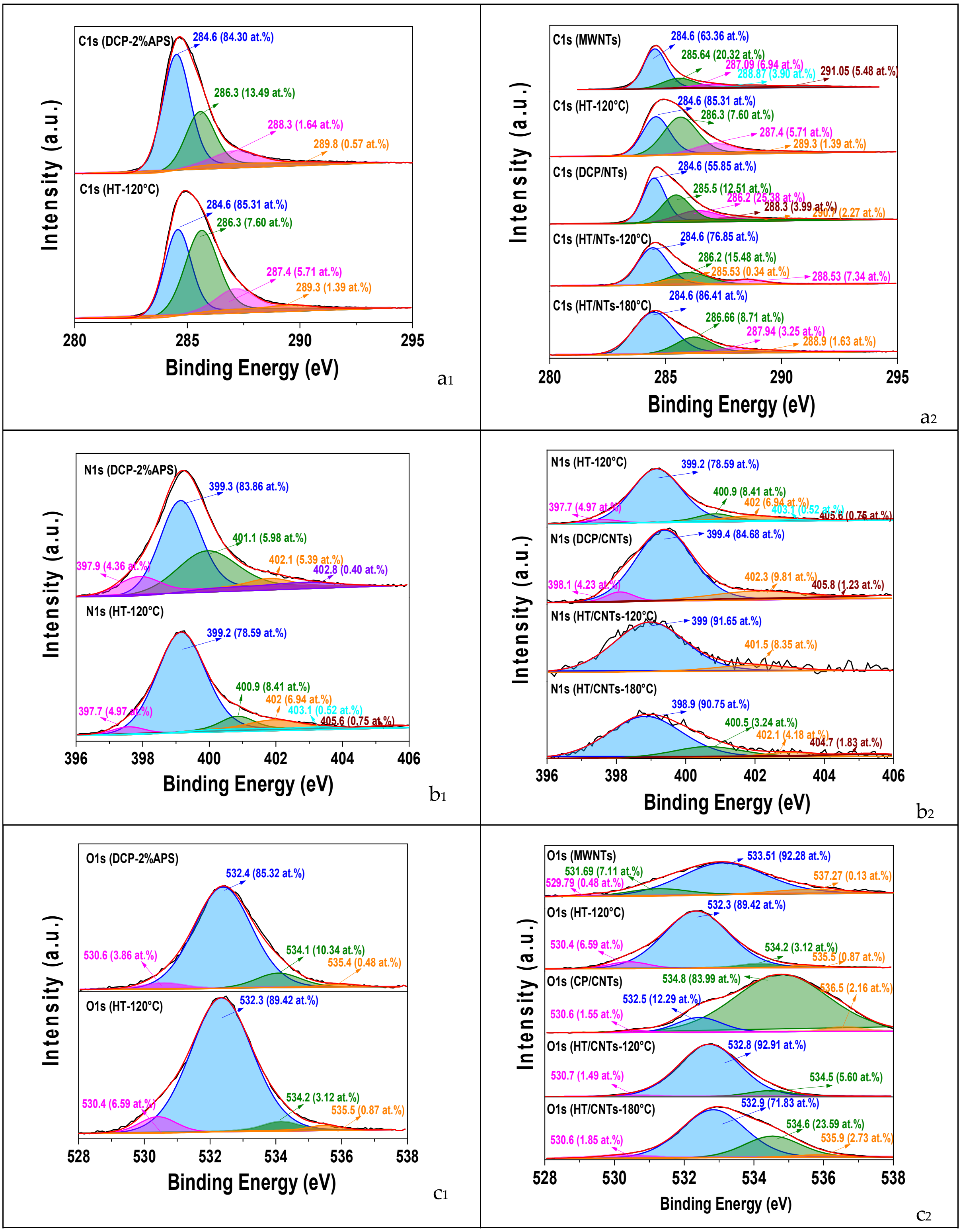

Figure S1. The deconvolution of the XPS C1S, N1s, and O1s spectra of pure PANI-NFs (a_1,b_1,c_1) and their nanocomposites with O-MWCNTs (a_2,b_2,c_2).

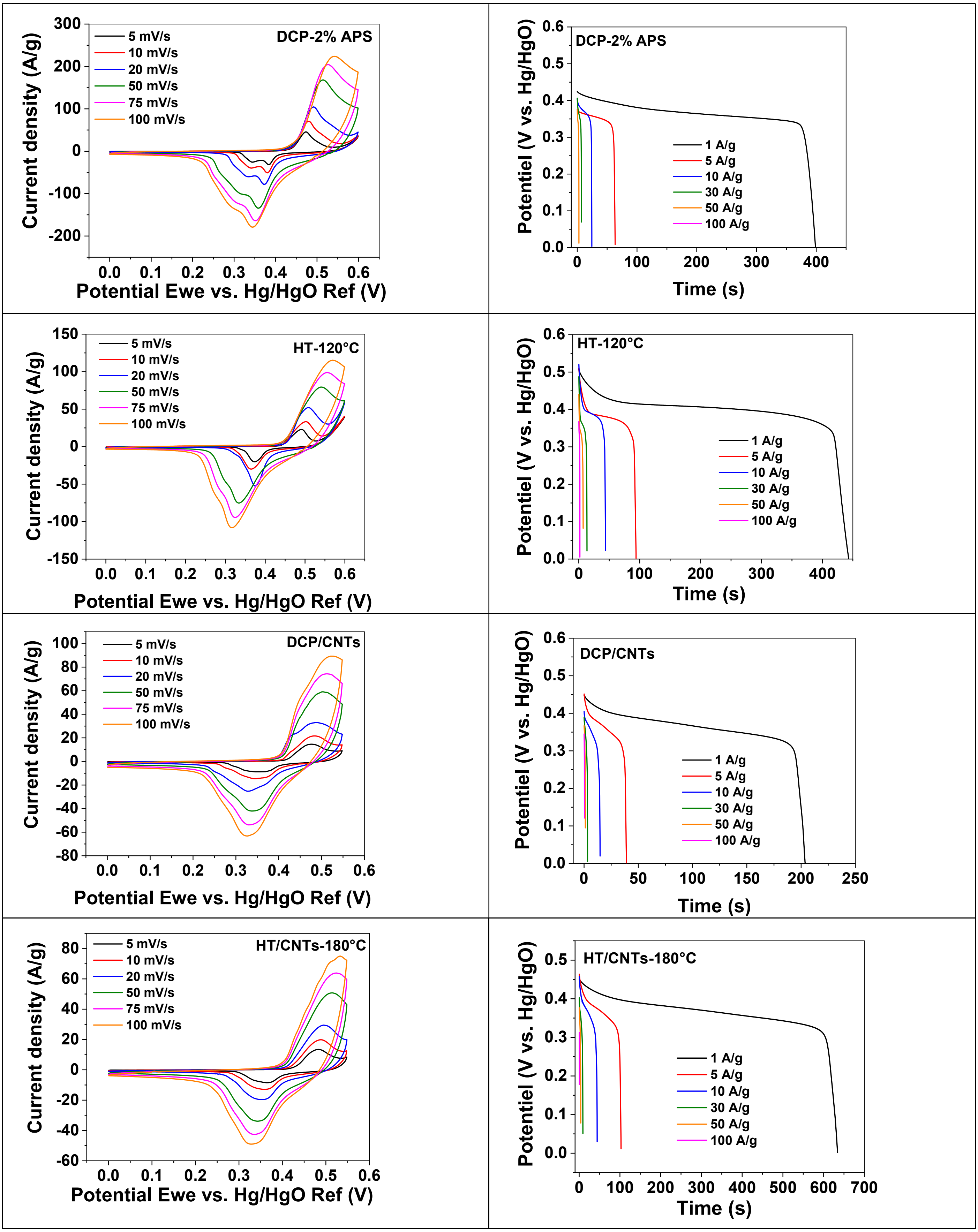

Figure S2. CV and GCD curves at different scan rates and current densities.

Table S1. The deconvolution of the XPS C1s spectra.

Sample → Binding ↓	DCP-2%APS	HT/120°C	O-CNTs	DCP/CNTs	HT/CNTs/120°C	HT/CNTs/180°C
C-C/C=C (B)	284.6<br>84.30 %	284.6<br>85.31%	284.6<br>63.36%	284.6<br>55.85%	284.6<br>76.85%	284.6<br>86.41%
C-N/C=N (Q)	-	-	-	285.54<br>12.51%	285.53<br>0.34%	-
C-O/C-O-C	286.33<br>13.49%	286.29<br>7.60%	285.64<br>20.32%	286.2<br>25.38%	286.21<br>15.47%	-
C-N+/C=N+	-	287.41<br>5.71%	287.1<br>6.94%	-	-	286.66<br>8.71%
C=O<br>O/C=O	288.25<br>1.64%	-	288.9<br>3.9%	288.25<br>3.99%	288.53<br>7.34%	287.94<br>3.25%
-COOH<br>O-COO<br>$\pi - \pi^*$	289.76<br>0.57%	289.34<br>1.38%	291.1<br>5.48%	290.06<br>2.27%	-	288.9<br>1.63%

Table S2. The deconvolution of the XPS O1s spectra.

Sample → Binding ↓	DCP-2%APS	HT/120°C	O-CNTs	DCP/CNTs	HT/CNTs/120°C	HT/CNTs/180°C
**O-C/C-OH**	**530.59<br>3.86%**	**530.41<br>6.59%**	**529.8<br>0.48%**	**530.62<br>1.55%**	**530.65<br>1.49%**	**530.62<br>1.85%**
**O=C**	**532.39<br>85.32%**	**532.34<br>89.42%**	**531.7<br>7.11%**	**532.51<br>12.30%**	**532.75<br>92.91%**	**532.90<br>71.83%**
**H_2O**	**534.08<br>10.04%**	**534.17<br>3.12%**	**533.5<br>92.3%**	**534.81<br>83.99%**	**534.49<br>5.60%**	**534.64<br>23.59%**
	**535.41<br>0.78%**	**535.45<br>0.87%**	**537.3<br>0.13%**	**536.46<br>2.16%**		**535.9<br>2.73%**

Table S3. The deconvolution of the XPS N1s spectra.

Sample → Binding ↓	DCP-2%APS	HT/120°C	DCP/CNTs	HT/CNTs/120°C	HT/CNTs/180°C
**-N= imine (Q)**	**397.85<br>4.36%**	**397.65<br>4.57%**	**398.1<br>4.28%**	**-**	**-**
**-NH-/-NH_2 amine (B)**	**399.25<br>83.67%**	**399.45<br>78.59%**	**399.37<br>84.68%**	**398.96<br>91.65%**	**398.94<br>90.80%**
**-N^+=/-NH^+-**	**401.05**	**400.85**	**-**		**400.5**

Bipolaran (Q^+)	5.98%	8.61%			3.64%
$-NH_2^+-$ Polaran (B^+)	402.09<br>5.59%	401.97<br>6.96%	402.34<br>9.81%	401.53<br>8.35%	402.1<br>4.18%
N^+ / ox	402.81<br>0.40%	403.09<br>0.52%	-	-	-
	-	405.57<br>0.75%	405.78<br>1.23%	-	404.74<br>1.38%

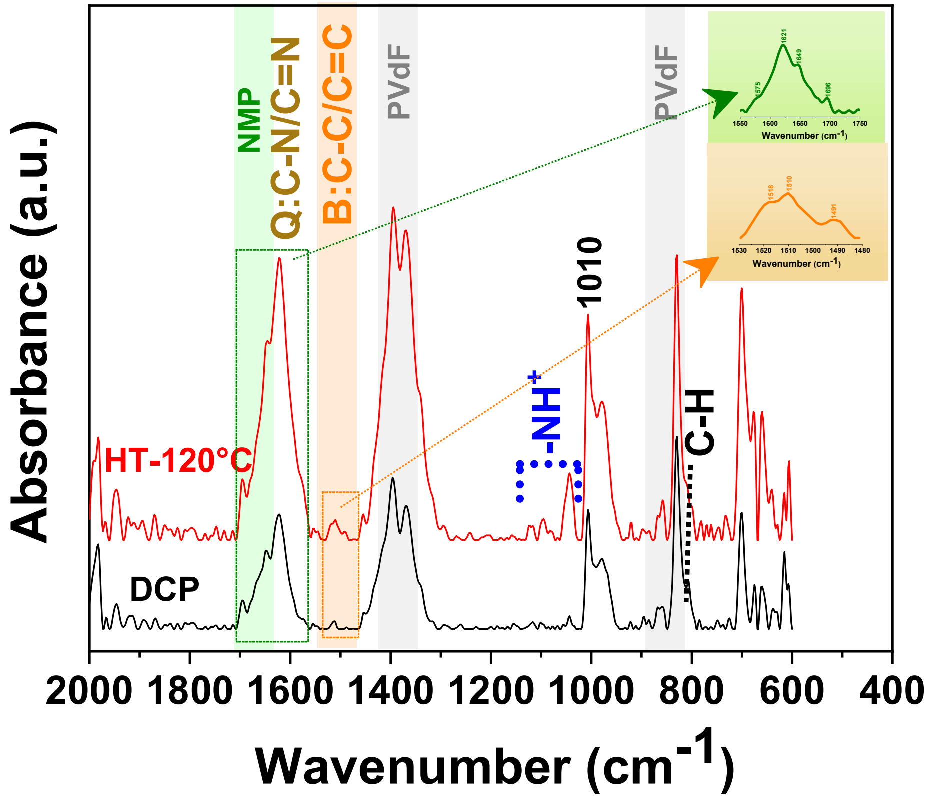

Figure S3. Structural stability of PANI-NFs after electrochemical cycling: FTIR spectra of doping retention and deprotonation effects.

To assess electrochemical stability, we analyzed the structural evolution of PANI-NFs after 10,000 GCD cycles in a 6M KOH electrolyte (Figure S3), confirming the retention of chemical structure and integrity post-testing, observing the ES ↔ EB transition and correlating it with supercapacitor performance. Moreover, for a polyaniline (PANI) electrode deposited on nickel foam (NiF) with PVDF binder, carbon black (CB) in NMP, the characteristic FTIR bands of the emeraldine salt (ES) and emeraldine base (EB) forms remain those of PANI, with contributions from PVDF, CB, and NMP generally distinguishable by their own distinct peaks.

Generally, during charge/discharge cycling in the KOH electrolyte, PANI undergoes redox transitions between leucoemeraldine ↔ emeraldine ↔ pernigraniline. In alkaline conditions,

this often leads to progressive deprotonation, shifting the conductive emeraldine salt (ES) form toward the less conductive emeraldine base (EB) or degraded products, directly impacting conductivity and capacitive performance over time. Over time, the polymer structure may oxidize or depolymerize, reducing the density of protonated sites, and thus, decreasing the intensity of the C–N$^+$ and polaronic bands (around 1230 and 1140 cm^{-1}). In other words, the more that cycling leads to the degradation or dedoping of PANI, the more that these characteristic bands weaken or shift, reflecting a loss in conductivity and electrochemical activity.

Figure S3 illustrates the structural evolution of PANI-NFs before and after electrochemical cycling, as revealed by FTIR spectroscopy, highlighting the retention of key functional bands and the deprotonation effects after prolonged cycling in KOH electrolyte. In this figure, the FTIR spectrum of the PANI/PVDF/CB electrode on Ni foam shows distinct contributions: PVDF exhibits strong peaks at 1400–1200 cm^{-1} and 840–880 cm^{-1}, assigned to the vibrational modes r(CH_2)-υ_a(CF_2), and w(CH_2)-υ_a(CC), respectively [1,2], confirming binder presence. Carbon black contributes a weak, broad band near 1600 cm^{-1}, assigned to the vibrational mode C=C, often masked by PANI's aromatic signals. Residual NMP may show the C=O and C–N stretching modes, situated at 1700–1650 cm^{-1} [3], and 1400–1000 cm^{-1} [4], but these should be minimal after drying. The aqueous KOH electrolyte has no significant direct FTIR signature; instead, its influence is observed indirectly through shifts in PANI's quinoid, benzenoid, and polaronic band intensities during redox processes.

As reported in the literature on PANI/Ni foam supercapacitors using a KOH electrolyte, we observe the same characteristic peaks of ES, sometimes with slight shifts (±5–20 cm^{-1}) depending on the dopant, morphology, and interactions with the Ni foam.

The goal is to correlate FTIR changes with electrochemical performance: after cycling, a drop in capacitance retention around 97% typically coincides with weakened C–N$^+$ bands (1140, 1230 cm^{-1}), indicating dedoping, and increased neutral benzenoid/quinoid bands, signaling a shift toward a less conductive emeraldine base (EB) or degradation. Conversely, stable performance correlates with well-preserved ES signatures, confirming structural integrity—thus, "capacity loss = loss of ES form", while "high retention = maintained protonated structure".

Therefore, post-cycling FTIR shows that PANI-ES bands are largely preserved, with the I_Q/I_B ratio increased to ~1.51 and oxidation degree x=0.6, indicating retained electroactivity. Peaks at 1045–1099 cm^{-1} confirm stable C–H stretching in benzenoid units. However, the DCP-2% sample lacks the 1100–1120 cm^{-1} polaron band (C–N$^{+\cdot}$/delocalized charge), while the HT-120 °C sample shows reduced intensity and slight shifts (±5–20 cm^{-1}), signaling partial deprotonation. The near absence of 1230–1250 cm^{-1} bands further supports this, yet ~97% capacitance retention suggests structural resilience, with minor conversion to a less conductive EB form—

more pronounced in PANI-NFs before hydrothermal treatment, correlating with slight performance loss.

References

1. Kobayashi, M.; Tashiro, K.; Tadokoro, H. Molecular vibrations of three crystal forms of poly(vinylidene fluoride). *Macromolecules* **1975**, *8*, 158e171. https://doi.org/10.1021/ma60044a013
2. Bachmann, M.A.; Koenig, J.L. Vibrational analysis of phase III of poly (vinylidene fluoride). *J. Chem. Phys*. **1981**, *74*, 5896e5910. DOI: 10.1063/1.440908
3. Lu, F.; Zhang, C.; Lu, B.; Yu, K.; Liu, J. ; Kang, H.; Liu, R.; Lan, G. Cellobiose as a model compound for cellulose to study the interactions in cellulose/lithium chloride/N-methyl-2-pyrrolidone systems. *Cellulose* **2017**, *24*, 1621–1629. DOI 10.1007/s10570-017-1213-1
4. McMurray, I.M.; Nettles, J.R.; Uzelmeier, A.W.; Swartz, J.A.; Newby, J.J. An analysis of the N-methyl-2-pyrrolidone: water complex using computational and matrix isolation FTIR methods. *J. Molec. Spectrosc*. **2025**, *408*, 111985. DOI: 10.1016/j.jms.2025.111985
